# Loss of the Volume-regulated Anion Channel Components LRRC8A and LRRC8D Limits Platinum Drug Efficacy

**DOI:** 10.1158/2767-9764.CRC-22-0208

**Published:** 2022-10-26

**Authors:** Carmen A. Widmer, Ismar Klebic, Natalya Domanitskaya, Morgane Decollogny, Denise Howald, Myriam Siffert, Paul Essers, Zuzanna Nowicka, Nadine Stokar-Regenscheit, Marieke van de Ven, Renske de Korte-Grimmerink, José A. Galván, Colin E.J. Pritchard, Ivo J. Huijbers, Wojciech Fendler, Conchita Vens, Sven Rottenberg

**Affiliations:** 1 Institute of Animal Pathology, Vetsuisse Faculty, University of Bern, Bern, Switzerland.; 2 COMPATH, Institute of Animal Pathology, Vetsuisse Faculty, University of Bern, Bern, Switzerland.; 3 Department of Radiation Oncology, The Netherlands Cancer Institute, Amsterdam, the Netherlands.; 4 Department of Biostatistics and Translational Medicine, Medical University of Lodz, Lodz, Poland.; 5 Mouse Clinic for Cancer and Aging Research (MCCA), Preclinical Intervention Unit, The Netherlands Cancer Institute, Amsterdam, the Netherlands.; 6 Translational Research Unit, Institute of Pathology, University of Bern, Bern, Switzerland.; 7 Mouse Clinic for Cancer and Aging Research (MCCA), Transgenic Facility, The Netherlands Cancer Institute, Amsterdam, the Netherlands.; 8 Department of Radiation Oncology, Dana-Farber Cancer Institute, Boston, Massachusetts.; 9 Department of Head and Neck Oncology and Surgery, The Netherlands Cancer Institute, Amsterdam, the Netherlands.; 10 Division of Molecular Pathology, The Netherlands Cancer Institute, Amsterdam, the Netherlands.; 11 Bern Center for Precision Medicine, University of Bern, Bern, Switzerland.; 12 Cancer Therapy Resistance Cluster, Department for BioMedical Research, University of Bern, Bern, Switzerland.

## Abstract

In recent years, platinum (Pt) drugs have been found to be especially efficient to treat patients with cancers that lack a proper DNA damage response, for example, due to dysfunctional BRCA1. Despite this knowledge, we are still missing helpful markers to predict Pt response in the clinic. We have previously shown that volume-regulated anion channels, containing the subunits LRRC8A and LRRC8D, promote the uptake of cisplatin and carboplatin in BRCA1-proficient cell lines. Here, we show that the loss of LRRC8A or LRRC8D significantly reduces the uptake of cisplatin and carboplatin in BRCA1;p53-deficient mouse mammary tumor cells. This results in reduced DNA damage and *in vivo* drug resistance. In contrast to *Lrrc8a*, the deletion of the *Lrrc8d* gene does not affect the viability and fertility of mice. Interestingly, *Lrrc8d^−^^/^^−^* mice tolerate a 2-fold cisplatin MTD. This allowed us to establish a mouse model for intensified Pt-based chemotherapy, and we found that an increased cisplatin dose eradicates BRCA1;p53-deficient tumors, whereas eradication is not possible in wild-type mice. Moreover, we show that decreased expression of *LRRC8A/D* in patients with head and neck squamous cell carcinoma, who are treated with a Pt-based chemoradiotherapy, leads to decreased overall survival of the patients. In particular, high cumulative cisplatin dose treatments lost their efficacy in patients with a low *LRRC8A/D* expression in their cancers. Our data therefore suggest that LRRC8A and LRRC8D should be included in a prospective trial to predict the success of intensified cisplatin- or carboplatin-based chemotherapy.

**Significance:**

We demonstrate that lack of expression of *Lrrc8a* or *Lrrc8d* significantly reduces the uptake and efficacy of cisplatin and carboplatin in Pt-sensitive BRCA1;p53-deficient tumors. Moreover, our work provides support to confirm the *LRRC8A* and *LRRC8D* gene expression in individual tumors prior to initiation of intensive Pt-based chemotherapy.

## Introduction

For over 40 years, platinum (Pt) compounds have been used as major components of chemotherapy regimens for several types of cancer (1). Even in the era of precision medicine and immunotherapy, Pt drugs remain among the most widely used anticancer drugs, due to their efficacy (2). Especially for cancer types in which the use of immune checkpoint inhibitors has been disappointing thus far, like ovarian cancer (3), Pt drugs are a mainstay of current therapy. Moreover, Pt-based chemotherapy may enhance the success of immunotherapy as shown for lung cancer as well as head and neck squamous cell carcinoma (HNSCC; refs. 4, 5). In Pt drug research, it is well established that DNA is the major cellular target (6). The three most frequently used Pt drugs in the clinic—cisplatin, carboplatin, and oxaliplatin—all affect normal DNA functions by generating monoadducts as well as intrastrand and interstrand DNA cross-links (2), subsequently leading to cell death in case these adducts remain unresolved. Consistent with this finding, Pt drugs synergize with tumors that show defects in the DNA damage response (7). A useful example is breast cancer. Pt drugs are not a standard treatment for breast cancer, because their efficacy on most forms of breast cancer is modest. However, patients with breast carcinomas that are defective in DNA repair by homologous recombination (HR) due to the lack of function of BRCA1, BRCA2, or other repair proteins in the HR pathway, do benefit from Pt-based chemotherapy (8–11). We also observed this in a genetically engineered *K14cre;Brca1^F/F^;Trp53^F/F^* (KB1P) mouse model for *BRCA1*-mutated breast cancer. The *Brca1^−^^/^^−^;Trp53^−^^/^^−^* mammary tumors were highly sensitive to cisplatin or carboplatin (12, 13).

Although HR deficiency scores provide useful information for patient stratification, the lack of additional reliable biomarkers for the prediction of Pt-based chemotherapy response still represents a major clinical limitation (2, 14). Despite the long use of Pt drugs, precision medicine approaches to tackle this challenge are still in their infancy. Moreover, those patients with disseminated tumors who show a major initial response usually develop secondary drug resistance. The precise mechanisms of Pt drug resistance remain poorly defined (2, 15, 16). One mechanism that has been confirmed in patients with *BRCA1-* or *BRCA2-*mutated cancers is the occurrence of secondary mutations in the *BRCA1* or *BRCA2* genes, leading to HR restoration (17, 18). Nevertheless, this mechanism alone does not explain all cases of secondary resistance (19) and it is less suitable for predicting upfront therapy response of patients with HR-defective tumors who receive their initial anticancer therapy. In the past, a major focus of the drug resistance studies was put on active Pt drug influx or efflux using tumor cell lines selected *in vitro* with Pt drugs. However, no transporter has been unambiguously linked to clinical Pt drug resistance thus far (15, 20).

In addition to transporters and diffusion, channels provide another route for Pt drugs to penetrate the cell membrane. Using genome-wide functional genetic screens for Pt drug resistance in haploid cells, we have identified volume-regulated anion channels (VRAC), composed of leucine-rich repeat containing (LRRC)8A and LRRC8D plasma membrane proteins, as the long sought-after plasma membrane entry points for cisplatin and carboplatin (21). VRACs, consisting of LRRC8 hexamers, contribute to the cellular volume regulation. The release of cellular solutes such as chloride and potassium ions reduces cell swelling under hypotonic conditions (22, 23). For successful formation of the hexameric channel structure at the plasma membrane, the subunit LRRC8A is obligatory, as its knockout (KO) abolishes chloride currents even when the other paralogs (LRRC8B-E) are overexpressed (22, 24). The composition of the other subunits (LRRC8B-E) is thought to determine the channel substrate specificity.

As the functional data on the role of LRRC8A and LRRC8D in Pt drug resistance are based on HR-proficient tumor cell lines thus far (21), we set out to study their role in the HR-deficient KB1P model. Indeed, loss of *Lrrc8a* or *Lrrc8d* in KB1P tumors largely abrogated the high Pt drug sensitivity of these tumors *in vitro* and *in vivo*, even though the uptake of cisplatin or carboplatin was reduced only by about 50% in LRRC8A- or LRRC8D-deficient *Brca1^−^^/^^−^;Trp53^−^^/^^−^* cells. Using *Lrrc8d^−^^/^^−^* mice, we show that intensified Pt therapy does eradicate KB1P tumors, whereas we are unable to do so in wild-type (WT) mice. Moreover, we corroborate the relevance of *LRRC8A* and *LRRC8D* gene expression for intensified Pt therapy in patients with HNSCC.

## Materials and Methods

### Lead Contact and Material Availability

Further information and requests for resources and reagents should be directed to and will be fulfilled by the lead contact, Sven Rottenberg (sven.rottenberg@vetsuisse.unibe.ch).

### Two-dimensional and Three-dimensional Cell Culture

For the two-dimensional (2D) cell culture, we used the cisplatin-sensitive, Brca1-mutated KB1PM5_control1 cell line, which we established previously (25). Cells were grown in DMEM/Nutrient Mixture F-12 (DMEM/F12; Gibco, Thermo Fisher Scientific, catalog no.10565018) supplemented with 10% FCS (Biowest, catalog no. S1810-500), 50 units/mL penicillin-streptomycin (Gibco, Thermo Fisher Scientific, catalog no. 15070063), 5 μg/mL insulin (Sigma, catalog no. I0516), 5 ng/mL cholera toxin (Sigma, catalog no. C8052), and 5 ng/mL murine EGF (Sigma, catalog no. E4127). Tissue culture of BRCA-deficient cell lines was carried out under low oxygen conditions (37°C, 5% CO_2_; 3% O_2_). Testing for *Mycoplasma* contamination was performed twice per year using PlasmoTest (InvivoGen, catalog no. rep-pt1).

The KB1P4N 3D tumor organoid line was previously established from a *Brca1^−/−^;Trp53^−/−^* mouse mammary tumor and cultured as described previously (26). Briefly, cultures were embedded in Cultrex Reduced Growth Factor Basement Membrane Extract Type 2 (BME; Trevigen Bio-Techne, catalog no. 3533-010010; 40 μL BME:growth media 1:1 drop in a single well of 24-well plate) and grown in Advanced DMEM/F12 (AdDMEM/F12, Gibco, catalog no. 11550446) supplemented with 1 mol/L HEPES (Sigma, catalog no. H0887), GlutaMAX (Gibco, catalog no. 35050061), 50 U/mL penicillin-streptomycin (Gibco, catalog no. A9165), B27 (Gibco, catalog no. 17504044), 125 μmol/L N-acetyl-L-cysteine (Sigma, catalog no. A9165), and 50 ng/mL murine EGF (Sigma, catalog no. E4127). Organoids were cultured under standard conditions (37°C, 5% CO_2_) and regularly tested for *Mycoplasma* contamination. Before transplantation, organoids were tested for pathogen contamination by IDEXX BioAnalytics services. For all cell cultures, testing for *Mycoplasma* contamination was performed twice per year using PlasmoTest (InvivoGen, catalog no. rep-pt1). 2D and three-dimensional (3D) KB1P cell lines were authenticated by a specific genotyping PCR for the introduced *Brca1* and *p53* mutations (25).

### Genome Editing, Plasmids, and Cloning

Generation of CRISPR/Cas9 plasmids was performed using a modified version of the pX330 backbone (Addgene, catalog no. 42230) into which a puromycin resistance open reading frame (ORF) was cloned under the hPGK promoter (27) for 2D cell lines or the lentiCRISPRv2 backbone (Addgene, catalog no. 52961, RRID:Addgene_52961) for the 3D organoids. The single-guide RNA (sgRNA) sequences (Supplementary Table S1) were cloned in the backbones using custom DNA oligos with the corresponding overhangs (Microsynth), which were melted at 95°C for 5 minutes, annealed at room temperature for 2 hours and subsequently ligated with quick ligase (NEB, catalog no. M2200S) into BbsI (NEB, catalog no. R0539) digested pX330 or BsmBI-digested (Fermentas, catalog no. FD0454) lentiCRISPRv2 backbone. Sanger sequencing verified the correctness of all constructs’ sequences.

LRRC8A- and LRRC8D*-*deficient 2D cell lines were generated by transfection with pX330 vectors containing gRNAs targeting the respective genes. In brief, KB1PM5 cells were transfected with 2.5 μg of plasmid DNA using the Mirus TransIT-LT1 reagent (Mirus, catalog no. MIR2300) and the corresponding protocol. Selection was performed using puromycin (Gibco, Thermo Fisher Scientific, catalog no. A1113802) at a concentration of 3 μg/mL for 72 hours after transfection. Monoclonal cell lines were isolated by dilution of single cells per well into 96-well plates. Clones bearing big deletion mutations, created by pairing sgRNAs to target the same gene, were identified by gel electrophoresis resolution of PCR amplicons corresponding to edited loci (amplicon primer sequences below). Sanger sequencing also confirmed the gene disruption. Amplicon primers for *Lrrc8a*: FW: 5′-ACAGAGCTCCGCTACTTTGC-3′ and RV: 5′-GGATGGTCACGTCGGGTATC-3′, amplicon primers for *Lrrc8d*: FW: 5′-CCCTTGCGGAAGTTGCTTCA-3′ and RV: 5′-CAGCTCCTGCTTATCCTGGG-3′.

### Genomic DNA Isolation, PCR Amplification, and Tracking of Indels by Decomposition Analysis

To determine the modification rate of cell populations which had been transfected/transduced using individual sgRNA targeting the gene of interest, the gene region was sequenced and analyzed using the Tracking of Indels by Decomposition (TIDE) tool. In brief, cells were pelleted and genomic DNA was extracted using the QIAmp DNA mini kit (Qiagen, catalog no. 51306) according to manufacturer's protocol. Target loci were amplified using Phusion High Fidelity Polymerase (Thermo Fisher Scientific, catalog no. F530L) using following conditions: (i) 98°C, 30 seconds, (ii) 30 cycles of 98°C for 10 seconds, 63.8 (*Lrrc8a*) or 64.2 (*Lrrc8d*) °C for 20 seconds, and 72°C for 30 seconds, (iii) 72°C, 5 minutes. The reaction mix consisted of 10 μK of 2x Phusion Mastermix 1 μL of 10 μmol/L forward and reverse primer and 100 ng of DNA in 20 μL total volume. PCR products were purified using the QIAquick PCR purification kit (Qiagen, catalog no. 28104) according to manufacturer's protocol. Subsequently, the PCR product was submitted to Sanger sequencing with the corresponding forward primers (listed below). Target modification rate was determined from the chromatogram (.ab1) sequence using the TIDE algorithm (28). For *Lrrc8a* sgRNA3 amplicon: FW: 5′-GGCCATTGGTGGGGTTCTTA-3′ and RV: 5′-GGTGCGTGGAAACTTGAACC-3′ (product size 550 bp), sequencing Primer: FW: 5′-ACAGAGCTCCGCTACTTTGC-3′, For *Lrrc8d* sgRNA2 amplicon: FW: 5′-AAAGGGTTCTCATTGGTCCCAC-3′ and RV: 5′-CGCCTTAGTTGTCCAGGGAG-3′ (product size 730 bp), sequencing primer: FW: 5′-AGGGAGGGCCAGATGGTAAC-3′.

### Reconstitution of *Lrrc8a/d* cDNA

*Lrrc8a* or *Lrrc8d* reconstitution was performed using a modified version of the pOZ-N-FH-IL2Rα plasmid (kindly provided by Dipanjan Chowdhury, Harvard Medical School, Boston, MA). Briefly, the vector backbone was amplified using the following primers, which excluded the FLAG and HA tags from the original vector from the linearized vector PCR product: FW: 5′-TCGAGAGATCCGGGAGACACAA-3′ and RV: 5′-CTCGAGCGGAAGATCTGGCAGTCT-3′. The *Lrrc8a* or *Lrrc8d* coding sequence was amplified from freshly prepared cDNA by primers including the C-terminal Myc sequence and corresponding plasmid overlaps suited for subsequent cloning using the in-fusion HD cloning kit by Takara Bio (Takara, catalog no. 12141). *Lrrc8a* FW: 5′-GATCTTCCGCTCGAGATGATTCCGGTGACAGAGCTCCGC**-**3′ and RV: 5′-TTGTGTCTCCCGGATCTCTCGATGCGGCCCTACAGATCCTCTTCTGAGATGAGTTTTTGTTCTCCTCCAGCGGCCGCGGCCTGCTCCTTGTCAGCTC-3′ *Lrrc8d* FW: 5′-GATCTTCCGCTCGAGATGTTTACCCTTGCGGAAGTTGC-3′ and RV: 5′-TTGTGTCTCCCGGATCTCTCGATGCGGCCCTACAGATCCTCTTCTGAGATGAGTTTTTGTTCTCCTCCAGCGGCCGCAATCCCGTTTGCAAAGGGGACA-3′. Correct cDNA insertion was verified by Sanger sequencing of the complete ORFs. A total of 50% confluent phoenix retrovirus producer cells (Gentaur Molecular Products, catalog no. RVK-1001, RRID:CVCL_H717) were transfected with pOZ‐*Lrrc8a*-Myc, pOZ-*Lrrc8d-*Myc or empty pOZ-Myc using Turbofectin transfection reagent (Origene, LabForce catalog no. TF81001). The next day, virus‐containing supernatant was collected, filtered through a 0.45 μm filter before application to LRRC8A- or LRRC8D-deficient monoclonal target cells. A total of 8 μg/mL Polybrene (Merck Millipore, catalog no. TR-1003-G) was added to each target cell dish. Virus was harvested and applied to target cells on three consecutive days. IL2Rα-expressing cells were selected using magnetic beads coated with a CD25 antibody (Dynabeads CD25; Thermo Fisher Scientific, catalog no. 11157D).

### Lentiviral Transduction of Organoids

Lentiviral stocks were generated by transient transfection of HEK293FT cells, which we obtained from Thermo Fisher Scientific (catalog no. R70007; RRID:CVCL_6911). On day 0, 8 × 10^6^ HEK293FT cells were seeded in 150 cm^2^ cell culture dishes and on the next day transiently transfected with lentiviral packaging plasmids and the plentiCRISPRv2 vector containing the respective sgRNA (*Lrrc8a* sgRNA 3 or *Lrrc8d* sgRNA2) or a nontargeting sgRNA using 2x HBS (280 nmol/L NaCl, 100 mmol/L HEPES, 1.5 mmol/L Na2HPO4, pH 7.22), 2.5 mol/L CaCl_2_ and 0.1× TE buffer (10 mmol/L Tris pH8.0, 1 mmol/L EDTA pH 8.0, diluted 1:10 with dH_2_O). After 30 hours, virus-containing supernatant was concentrated by ultracentrifugation at 20,000 rpm for 2 hours in a SW40 rotor at 4°C and the virus was finally resuspended in 100 μL PBS. The virus titer was determined using a qPCR Lentivirus Titration Kit (Applied Biological Materials, catalog no. LV900). Tumor-derived organoids were transduced according to a previously established protocol (26). The target sites modifications of the polyclonal cell pools were analyzed by TIDE analysis as described previously.

### Clonogenic Assays

For clonogenic growth assays in a 6-well plate (TPP, catalog no. 92406) format, 2,000 KB1PM5 cells were seeded per well in DMEM-F12 complete medium. A total of 24 hours after seeding, the cells were treated with the indicated drug doses over the course of 24 hours. After 8 days, the wells were fixed with 4% paraformaldehyde (PFA)/PBS and stained with 0.1% crystal violet. Quantification of the wells was performed with ImageJ using the ColonyArea plugin (version 1.53i; ref. 29).

### Growth Assays

For growth assays, KB1PM5 cells were seeded to 96-well plates (TPP, catalog no. 92696) at a density of 100 cells per well. Proliferation was measured on 7 consecutive days using CellTiter-Blue Cell Viability Assay (Promega, catalog no. G9241) following manufacturer's instructions.

### Competition Assays

The polyclonal cell pools generated by transfection with individual sgRNAs targeting either *Lrrc8a* or *Lrrc8d* were used for competition assays. In brief, cells were seeded to 12-well plates (TPP, catalog no. 92412) at 1,000 cells/well and drug selection using different Pt-based agents was applied at indicated concentrations for 24 hours. After 8 days of recovery, cells were either fixed with PFA or stained with crystal violet (Supplementary Fig. S3D) or harvested and the gDNA isolated and processed for subsequent TIDE analysis as described previously (28). The shift of mutated alleles was determined by comparing the modification rates of drug naïve and drug selected polyclonal cell populations.

### Western Blotting

Cells were washed and scraped in cold PBS. After pelleting, cells were lysed in RIPA buffer (50 mmol/L Tris-HCl pH 7.4; 1% NP-40; 0.5% Na-deoxycholate; 0.1% SDS; 150 mmol/L NaCl, 2 nmol/L EDTA, 50 mmol/L NaF) containing 1x complete protease inhibitor cocktail (Roche, catalog no. 04693132001) for 60 minutes on ice, followed by homogenization via syringe and needle. The lysate was subsequently cleared by centrifugation for 10 minutes at 14,000 rpm. Protein concentrations of the supernatants were determined using the Pierce BCA assay kit (Thermo Fisher Scientific, catalog no. 23225) with a BSA standard curve. Protein lysates were denatured at 70°C for 10 minutes in SDS sample buffer [Laemmli SDS sample buffer, reducing (6x); Thermo Fisher Scientific, catalog no. J61337.AC] and separated by SDS-PAGE on 7.5% acrylamide gels before overnight wet transfer for 18 hours at 15 V to 0.45 μm pore size polyvinylidene difluoride membranes (GE Healthcare, catalog no. 10600018). Membranes were blocked in 5% BSA in Tris-buffered saline with Tween-20 (TBS-T, 100 mmol/L Tris, pH 7.5, 0.9% NaCl, 0.05% Tween-20) and subsequently incubated with primary antibodies diluted 1:1,000 (anti-LRRC8A rabbit polyclonal, Bethyl Laboratories, catalog no. A304-175A, RRID:AB_2621424, anti-LRRC8A, anti-LRRC8D rabbit polyclonal provided by T. Jentsch) or 1:2,000 (anti-beta Actin, mouse monoclonal Sigma, catalog no. A1978, RRID: AB_476697 and anti-alpha tubulin, mouse monoclonal Sigma, catalog no. T5168, RRID: AB_477579) in blocking buffer at 4°C overnight. After washing in TBS-T, horseradish peroxidase (*HRP*)-linked secondary antibodies diluted 1:2,500 (anti-mouse IgG and anti-rabbit IgG, Cell Signaling Technology, catalog no. 7076, RRID:AB_330924, and catalog no. 7074, RRID:AB_10684258, respectively) were applied for 2 hours at room temperature. Images were acquired using the FUSION FX7 imaging system (Vilber GmbH).

### *In Vivo* Validation of Resistance

All animal experiments were approved by the Animal Ethics Committee of The Netherlands Cancer Institute (Amsterdam, the Netherlands) and the Animal Ethics Committee of the canton of Bern (Switzerland) and are in accordance with the current Dutch and Swiss Acts on Animal Experimentation. CRISPR-Cas9–modified organoid lines derived from *K14cre; Brca1F/F;Trp53F/F* (KB1P) female mice were transplanted in 6–9 weeks old NU/J nude mice (strain ID 3484) for the *in vivo* validation. The *Lrrc8a* or *Lrrc8d* modification rate in the outgrown tumors (*N* = 6 each) was determined by TIDE analysis as described previously. Tumors with high modification rate percentage were chosen for the *in vivo* validation experiments.

For tumor piece transplantation, DMSO-frozen tumor pieces were thawed, washed with PBS, cut into small pieces and transplanted in the fourth right mammary fat pad of 6–9 weeks old NMRI nude mice. Mammary tumor size was measured by caliper measurements and tumor volume was calculated (length × width^2^/2). Animals were randomly assigned to the treatment groups. Treatment of tumor-bearing mice was initiated, when tumors reached a size of approximately 75 mm^3^. Carboplatin (Teva, catalog no. 6985451) was administered at 50 mg/kg intravenously over the course of two cycles (14 days between treatments). Animals were sacrificed with CO_2_, when the tumor reached a volume of 1,500 mm^3^. Animal technicians who were blinded regarding the hypothesis performed tumor size measurements and treatments.

### Generation of *Lrrc8d* KO Mice

The *Lrrc8d* conditional KO mice were generated by an established CRISPR/Cas9-mediated gene editing protocol (30). Briefly, LoxP sites were introduced to flank the first coding exon of the *Lrrc8d* gene sequence (exon 3). Zygotes isolated from FVB mice were coinjected with a microinjection mix containing *in vitro* transcribed Cas9 mRNA, two sgRNAs targeting the different sites in the *Lrrc8d* gene (5′–CACCGGCTTCAGGATTCGGTAAGT-3′ and 5′–CACCGCAGGCACACCCACGTGCGG-3′) and two homology-directed repair oligos, each containing a LoxP site. Zygotes that have further divided into two-cell embryos after overnight incubation at 37°C, 5% CO_2_, were surgically implanted into the oviduct of a pseudopregnant foster mother. Correct incorporation of the LoxP sites was confirmed by PCR. Subsequently, these mice were crossed with Cre recombinase expressing FVB mice, resulting in offspring with a 2,561 bp deletion in exon 3 of the *Lrrc8d* gene. Gene disruption was confirmed by PCR and the decrease in *Lrrc8d* expression levels was determined by qRT-PCR and Western blotting. Primer for *Lrrc8d* deletion genotyping PCR: FW: 5′-TTTCAGGAATGTTTACCCTTGCGG-3′ and RV: 5′-TGCATCGTGTCCTGTTTAAAGGGC-3′ (PCR product size if positive: 156 bp), Primer for WT PCR: FW: 5′-TTTCAGGAATGTTTACCCTTGCGG-3′ and RV: 5′-GGTGTGTGGCTGTTTCCATCCTG-3′ (PCR product size if positive: 270 bp).

### qRT-PCR of *Lrrc8d* Mice

For the expression determination of *Lrrc8d* using qRT-PCR, a mastermix containing FastStart Universal SYBR Green Master Mix (Roche, catalog no. 4913850001), forward and reverse primers at a final concentration of 300 nmol/L and water was prepared. A total of 11 μL of this mastermix were mixed with 4 μL of cDNA template dilution (final dilution of 5 ng/μL of cDNA in reaction) in a MicroAmp Fast Optical 96-Well Reaction Plate (Thermo Fisher Scientific, catalog no. 4343906) to subsequently be subjected to following PCR program: (i) 95°C, 10 minutes, (ii) 40 cycles of 95°C for 10 seconds, 58°C 30 seconds, melting curve on a ABI 7500Fast device (Thermo Fisher Scientific catalog no. 4406985). Expression levels are displayed relative to the *Hprt* housekeeping gene control expression. *Lrrc8d* Primer pair 1: FW: 5′-CTG CCT CTA CAC TCT CTT CTG GC-3′ and RV: 5′-CGC AAA GTC GTT CTT GAC ATC CG-3′. *Lrrc8d* Primer pair 2: FW: 5′-CTG ACA TAC CTC TCC AAG CCA CC-3′ and RV: 5′-GTC TCT CTT CTC CTT CTT CGC CT-3′. *Hprt* Primer pair: FW: 5′-CCT AAG ATG AGC GCA AGT TGA A-3′ and RV: 5′-CCA CAG GAC TAG AAC ACC TGC TAA-3′.

### LRRC8D Protein Levels in Mouse Kidneys

To determine the LRRC8D protein levels in WT, heterozygous, and homozygous KO mice, protein was isolated from whole kidneys by metal bead homogenization in PBS and subsequent lysis in T-PER Tissue Protein Extraction Reagent (Thermo Fisher Scientific, catalog no. 78510) and 1x Halt protease inhibitor cocktail (Thermo Fisher Scientific, catalog no. 87786). A total of 80 μg protein was subjected to electrophoresis and Western blotting. Antibody incubations using the rabbit anti-LRRC8D polyclonal antibody provided by T. Jentsch and subsequent imaging were performed as described previously.

### Cisplatin Adduct and yH2AX IHC Staining of Mouse Kidneys After Treatment

Littermate WT, heterozygous, and homozygous *Lrrc8d* KO mice were intravenously treated with 6 mg cisplatin per kg (Teva, catalog no. 4333164). After 6 hours, the animals were anesthetized with isoflurane, sacrificed with CO_2_ followed by organ harvest. Kidneys of vehicle- or cisplatin-treated mice were fixed in 4% PFA and further embedded in paraffin. Each paraffin block was sectioned at 2.5 μm and IHC was performed on an automated immunostainer (Leica Bond RX, Leica Biosystems, catalog no. 95735-848538). The following antibodies were used for IHC staining of tumors: the homemade NKI-A59 antibody for the detection of cisplatin DNA adducts as described previously (31) and the Phospho-Histone H2A.X Ser139 20E3 Rabbit mAb (Cell Signaling Technology, catalog no. 9718, RRID:AB_2118009) for the detection of yH2AX foci. Then all samples were incubated with HRP polymer for 15 minutes and subsequently visualized using 3,3′-diaminobenzidine as a brown chromogen (Bond polymer refine detection, Leica Biosystems, catalog no. DS9800, RRID:AB_2891238) for 10 minutes. The samples were counterstained with hematoxylin for 5 minutes, dehydrated and mounted with Pertex (Sakura). Slides were scanned on a Panoramic 250 Flash scanner (3DHISTECH).

The percentage of positive nuclei in 50 kidney cortex image sections per kidney (for NKI-A59 antibody) using ImageJ (version 1.53i) or the whole cortical kidney region of each mouse (for yH2AX) determined by QuPath v0.3.0 were quantified and normalized via the average basal levels of the vehicle treated samples. Numbers of animals per group, vehicle: wt *N* = 5, het KO *N* = 8, hom KO *N* = 8; treated: wt *N* = 8, het KO *N* = 12, hom KO *N* = 16. The data represent the normalized mean percentage of positive nuclei of all the image sections per mouse ± SD (two-way ANOVA, followed by Tukey multiple comparisons test). A pathologist who was blinded concerning the sample identity carried out the quantification of both stainings.

### Imaging Mass Cytometry Measurement of Pt Content in the Kidneys

The same kidney samples as for the Pt-adduct antibody IHC stainings were used for the imaging mass cytometry (IMC) analysis. Three kidneys of either cisplatin-treated or untreated WT or homozygous LRRC8D-deficient mice underwent direct Pt-content analysis. Briefly, formalin-fixed paraffin-embedded sections of 3.5 μm thickness on Superfrost Plus slides were dewaxed by Xylol and rehydrated stepwise by 100%, 94%, and 70% ethanol. Samples were washed with PBS and incubated with a 1:100 dilution of 25 μmol/L Ir-Intercalator (Fluidigm, catalog no. 201192A) for 30 minutes at room temperature to stain the nuclei. The samples were dipped three times in distilled water and air dried before IMC analysis. The stained and dried samples were then inserted into the HyperionTM Imaging System [Standard BioTools (formerly Fluidigm), evolved from that described by Giesen, and colleagues (32)], where the tissue was ablated by a 1 μm diameter UV laser. Tissue from an ablation spot was vaporized with each laser shot, and the plume containing the heavy metals present in the tissue was transported into the inductively coupled plasma ion source for detection. All data were acquired using the CyTOF software version 7.0.8493. Three 1 mm^2^ sections per kidney were acquired. For each image, the mean ^194^Pt, ^191^Ir, and ^134^Xe^+^ values of three image sections were quantified. The mean ^194^Pt values were subsequently normalized by the background ^134^Xe^+^ signal as described by Chang and colleagues (33). The data represent the normalized mean ^194^Pt signal of the image sections ± SD (two-way ANOVA, followed by Tukey multiple comparisons test).

### Drug Toxicity in *Lrrc8d* KO Mice

To determine the tolerability of higher cisplatin doses, littermate WT and homozygous *Lrrc8d* KO mice were subjected to 6, 9, or 12 mg/kg cisplatin i.v. on days 0 and 14. The body weight and physical state of the mice were monitored daily. The groups consisted of 5 animals each. Animals, where the bodyweight decreased below 90% of the starting weight before treatment were euthanized.

Cisplatin-resistant KB1P tumors were generated by repeated intraperitoneal administration of 4 mg cisplatin per kg to naïve KB1P tumors transplanted into FVB mice until no more treatment response was observed. This resulted in stably cisplatin-resistant tumors. To test whether increased cisplatin doses could eradicate naïve and resistant tumors, tumor pieces were transplanted to the fourth mammary fat pat of FVB mice as described previously. The cisplatin naïve and resistant tumors were treated intravenously with two cycles of cisplatin starting from a size of approximately 150 mm^3^ (days 0 and 14). Tumor sizes were monitored for the succeeding 150 days after treatment. Animals with tumors reaching a volume of 1,000 mm^3^ were sacrificed with CO_2_. For the treatment with 12 mg/kg of cisplatin homozygous *Lrrc8d* KO mice were used. Cisplatin naïve tumor groups: vehicle *N* = 5, 6 mg/kg *N* = 10, 12 mg/kg *N* = 5. Cisplatin resistant tumor groups: vehicle *N* = 5, 6 mg/kg *N* = 5, 12 mg/kg *N* = 5. Animal technicians who were blinded for the hypothesis of the treatment outcome conducted the tumor size measurements and treatments.

### Drug Uptake Measurement (CyTOF)

KB1PM5 *Lrrc8a/d* WT and KO cell lines were seeded 2 days prior to drug treatment. It was aimed to have a starting cell number of 300,000 cells per condition at a density of 80% on treatment day. On the day of treatment, drug-containing medium was freshly prepared with the indicated concentrations and cells were treated for 3, 6, or 24 hours. After the treatment, cells were washed three times with serum containing culture medium for 5 minutes. Subsequently, cells were washed with room temperature PBS and then incubated with 0.25% Trypsin EDTA. Trypsinization was stopped with serum containing culture medium and the cells were then fully dissociated into a single-cell suspension by gentle pipetting. After dissociation, the cells were counted and 300,000 cells per condition were used for further fixation and barcoding according to the Cell-ID 20-plex Pd Barcoding kit (Fluidigm, catalog no. 201060) protocol. Barcoded samples were then pooled and incubated with the Cell-ID intercalator Ir (Fluidigm, catalog no. 201192A) at 100 μL/1 Mio cells for 1 hour at room temperature and then stored at −80°C until measurement. For the measurement, samples were thawed, washed, mixed with equilibration beads and acquired on a Helios mass cytometer (Fluidigm). Postacquisition, data were bead-normalized and debarcoded using the premessa R package released by the Parker Institute for Cancer Immunotherapy (https://github.com/ParkerICI/premessa). Absolute Pt atom counts were determined by gating for Iridium^191^ + BeadDist and ^191^Iridium + ^193^Irridium events to exclude cell debris, and ^191^Iridium + event length to exclude duplet signals using FlowJo version 10.8.1. Median Pt counts for the isotopes ^192^Pt, ^194^Pt, ^195^Pt, ^196^Pt, and ^198^Pt were summed to determine the total amount of Pt atoms per cell. Data of a total of three independent replicates consisting of three technical replicates each, where approximately 100,000 cells were acquired per condition are shown for Fig. 2A. For Fig. 2B and C, three independent replicates, where approximately 50,000 cells were acquired per condition and replicate are shown. Statistical analysis was performed using GraphPad Prism 9 (two-way ANOVA followed by Tukey multiple comparisons test).

### Immunofluorescence

For the cisplatin-DNA-adduct staining using the homemade NKI-459 antibody, 60,000 cells were seeded to 12 mm diameter coverslips (thickness no. 1; Paul Marienfeld GmbH, catalog no. 0111520). After 24 hours, they were treated for the indicated durations with 10 μmol/L cisplatin. After treatment, the cells were washed with PBS and fixed with 4% PFA/PBS for 20 minutes at 4°C. Fixed cells were permeabilized for 20 minutes in 0.5% Triton X‐100/PBS, the DNA was denatured using 2 mol/L HCL for 10 minutes at 37°C. After washing with PBS, the cells were blocked in 1% BSA in PBS-T. The NKI-A59 antibody, diluted 1:200 in 1% BSA in PBS-T was applied for 2 hours at room temperature. The secondary antibody Alexa Fluor 488 goat anti-rabbit IgG (Thermo Fisher Scientific, catalog no. A-11034, RRID: AB_2576217) was diluted 1:2,000 in blocking buffer and incubated for 1 hour at room temperature. The DNA was stained with DAPI (Life Technologies, catalog no. D1306, 1:50,000 dilution) before mounting onto positively charged microscopy slides using fluorescent mounting medium (Dako, catalog no. S3023). Analysis was performed on a DeltaVision Elite High Resolution Microscope system (GE Healthcare) consisting of an Olympus IX‐70 inverted microscope with a CMOS camera, 100 × Olympus Objective, and Softworx (Applied Precision) software. Per condition and replicate, approximately 100 cells from different areas of the coverslips were imaged in Z-stacks of 21 slices of 0.2 μm thickness each. Images were analyzed using the FIJI image processing package of ImageJ (version 1.8.0; ref. 34). Briefly, Z-stacks of the individual channels were projected using the "sum slices" projection. All nuclei were detected by the “analyze particles” command using the DAPI channel. This region of interest (ROI) selection was then used to determine the raw integrated density of the NKI-A59 antibody staining of each nucleus. ROI touching the edges of the images were excluded. Data were plotted in GraphPad Prism software 9 and significance was calculated using two-way ANOVA followed by Tukey multiple comparisons test.

For the yH2AX foci detection, the cells were seeded to coverslips and treated with 2 μmol/L cisplatin for the indicated timepoints. After treatment, cells were washed with PBS and fixed with 4% PFA/PBS for 20 minutes at 4°C. Fixed cells were permeabilized for 20 minutes in 0.5% Triton X‐100/PBS. All subsequent steps were performed in staining buffer [PBS, BSA (2%), glycine (0.15%), Triton X-100 (0.1%)]. Cells were washed three times and blocked for 30 minutes at room temperature using the described staining buffer. The cells were incubated overnight at 4°C with the primary antibody anti-phospho-Histone H2A.X (ser139) (clone JBW301, Merck Millipore, catalog no. 05-636, RRID: AB_309864) at a dilution of 1:200 in staining buffer, washed three times and subsequently incubated with the secondary antibody Goat anti-Mouse IgG (H+L) Cross-Adsorbed Secondary Antibody Alexa Fluor (Thermo Fisher Scientific, catalog no. A-11029, RRID: AB_2534088) for 2 hours at room temperature. The coverslips were washed five times after secondary antibody staining, counterstained with DAPI as described previously and mounted onto positively charged Superfrost Plus Adhesion Microscope Slides (epredia, catalog no. J1800AMNZ) using fluorescence mounting medium (Dako, catalog no. S3023). Analysis was performed on a DeltaVision Elite High Resolution Microscope system (GE Healthcare) consisting of an Olympus IX‐70 inverted microscope with a CMOS camera, 100 × Olympus Objective, and SOFTWORX (Applied Precision) software. Per condition and replicate, approximately 200 cells from different areas of the coverslips were imaged in Z-stacks of 31 slices of 0.2 μm thickness. Images were analyzed using the FIJI image processing package of ImageJ (version 1.8.0; ref. 34). Briefly, Z-stacks of the individual channels were projected using the "max intensity" projection setting. All nuclei were detected by the “analyze particles” command using the DAPI channel projection and this ROI selection was then used to determine the number of yH2AX foci of each nucleus by the "finding maxima" command on the FITC channel. ROI touching the edges of the images were excluded. Data were plotted in GraphPad Prism 9 software and significance was calculated using ordinary one-way ANOVA followed by Tukey multiple comparisons test.

### Statistical Analysis HNSCC

The collection of the HNSCC biopsies was approved by the Institutional Review Board of the Netherlands Cancer Institute. All patients signed an informed consent for the collection and analysis of the HNSCC samples and the study was conducted in accordance with International Ethical Guidelines for Biomedical Research Involving Human Subjects (CIOMS). CNV data of *LRRC8A* or *LRRC8D* were obtained by shallow DNA sequencing of HNSCCs dataset (35). Gene expression data were obtained by polyA RNA sequencing (RNA-seq; ref. 36). Patients were classified into low or high *LRRC8A* expression by the cutoff at 20 rpkm.

### Statistical Analysis Ovarian Cancer

The GSE32063 dataset consists of 40 advanced-stage high-grade serous ovarian cancer samples. This study was performed after approval by the Institutional Review Board. All patients provided a written informed consent for the collection and analysis of these samples and the study was conducted in accordance with International Ethical Guidelines for Biomedical Research Involving Human Subjects (CIOMS). All patients were treated with a combined platinum-taxane standard chemotherapy. Gene expression levels were obtained by whole human genome microarray sequencing (Agilent-014850 4X44K G4112F; ref. 37). Patients were classified into low or high *LRRC8A* or *LRRC8D* expression by a cutoff of 33% (lower and upper tertile).

### Data Availability Statement

The HNSCC dataset was previously published by Essers and colleagues (35). The Dutch multicenter cohort CNA and RNA-seq data that support the findings of this study are available in the European Genome-Phenome Archive (EGA) at https://ega-archive.com under the EGA study numbers EGAS00001004090 (RNA-seq data), EGAS00001004091 [low-coverage whole-genome sequencing (WGS)] and dataset numbers EGAD00001005716, EGAD00001005715 and EGAD00001005719, EGA D00001005718 for the RNA-seq and low-coverage WGS, respectively.

The expression data of the ovarian cancer dataset were previously published by Yoshihara and colleagues and is available from Gene Expression Omnibus data repository by the accession number GSE32063 (37).

## Results

### Loss of *Lrrc8a* or *Lrrc8d* Induces Cisplatin and Carboplatin Resistance in BRCA1;p53-deficient Mouse Mammary Tumor Cells

**FIGURE 1:**
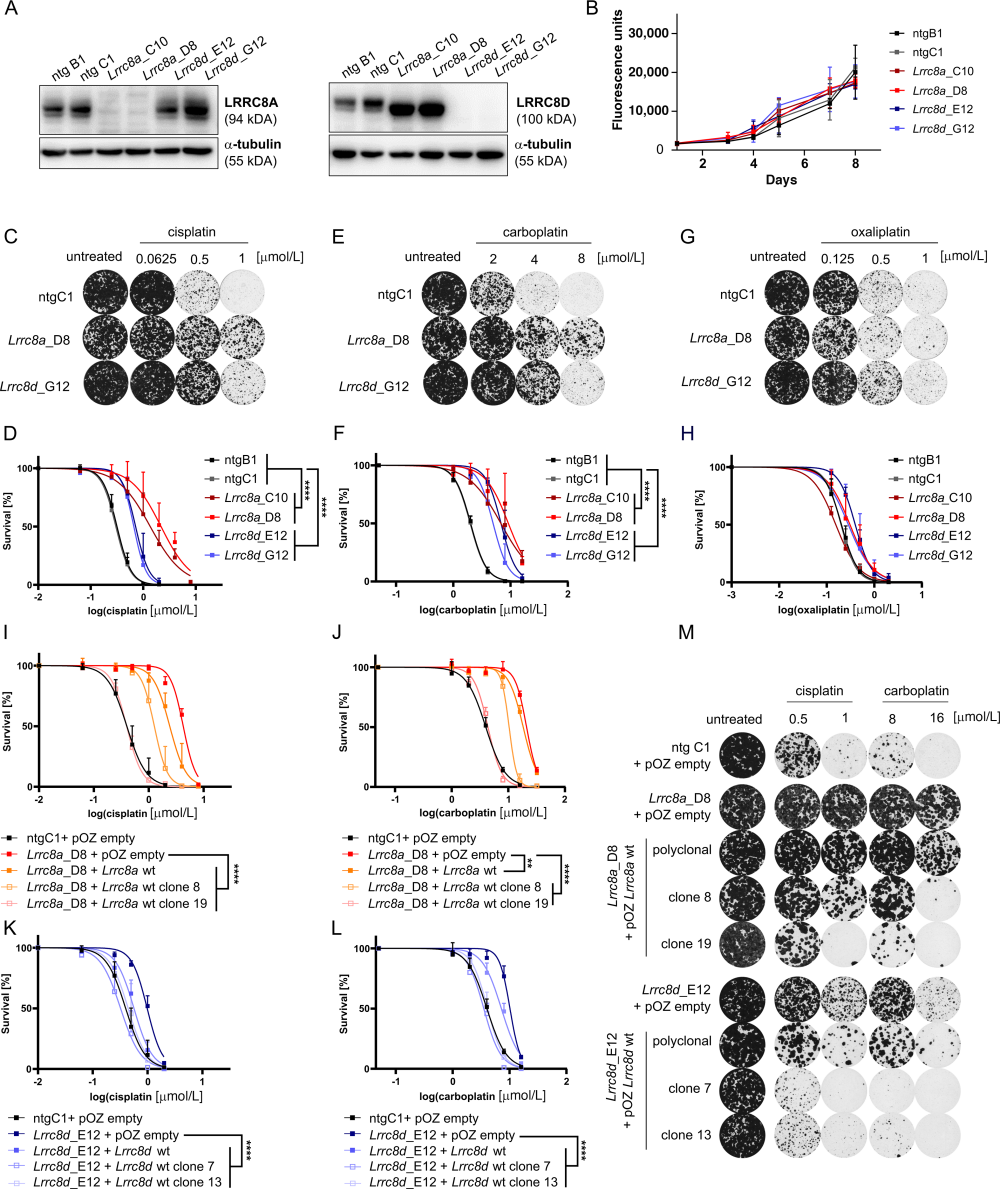
Loss of *Lrrc8a* or *Lrrc8d* induces Pt-drug resistance in BRCA1;p53-deficient mammary tumor cells. **A,** Representative Western blots of control (ntg B1 and ntg C1), *Lrrc8a* (*Lrrc8a*_C10 and *Lrrc8a*_D8), and *Lrrc8d* (*Lrrc8d*_E12 and *Lrrc8d*_G12) KO cell lines used in the validation experiments. **B,** Proliferation rates of WT and *Lrrc8a* or *Lrrc8d* KO cells. Mean ± SD of six replicates is shown. Clonogenic survival assays and quantification of WT and LRRC8A/D-deficient cell lines treated with cisplatin (**C** and **D**), carboplatin (**E** and **F)**, or oxaliplatin (**G** and **H**). Representative images of selected lines and concentrations are shown. Data represent mean ± SD of three independent replicates and were fitted to a four parameter logistic sigmoidal curve. *P* values are calculated by one-way ANOVA followed by Tukey multiple comparisons test for the log(IC_50_) values of the survival curves ****, *P* < 0.0001. **I–L,** Quantification of clonogenic growth assays using the different *Lrrc8a* (*Lrrc8a*_D8 + pOZ empty, + pOZ *Lrrc8a* wt polyclonal or clonal lines) or *Lrrc8d* (*Lrrc8d*_D8 + pOZ empty, + pOZ *Lrrc8d* wt polyclonal or clonal lines) rescue cell lines treated with cisplatin or carboplatin. As negative controls, empty vector-transduced cell lines were used. Data represent mean ± SD of three independent replicates and were fitted to a four parameter logistic sigmoidal curve. *P* values are calculated by one-way ANOVA followed by Tukey multiple comparisons test for the log(IC_50_) values of the survival curves. ****, *P* < 0.0001; **, *P* < 0.01. **M,** Representative images of selected conditions of the clonogenic growth assays in the presence of cisplatin or carboplatin, using the rescue cell lines from **I–L** are shown.

To investigate the effects of LRRC8A or LRRC8D defects on Pt drug sensitivity in HR-deficient tumors, we generated CRISPR/Cas9 KOs in cell lines derived from a genetically engineered mouse model for hereditary *BRCA1*-mutated breast cancer (25). Because of the irreversible *Brca1* deletion these cells are highly sensitive to Pt drugs and thereby provide a useful tool to study mechanisms of Pt drug resistance that are independent of a restoration of BRCA1 function (38). Using a paired gRNA approach to generate big deletions in the *Lrrc8a* or *Lrrc8d* genes (Supplementary Fig. S1A and S1B), we obtained monoclonal cell lines that lost expression of LRRC8A (KB1PM5-*Lrrc8a^−^^/^^−^*_C10, KB1PM5-*Lrrc8a^−^^/^^−^*_D8) or LRRC8D (KB1PM5-*Lrrc8d^−^^/^^−^*_E12, KB1PM5-*Lrrc8d^−^^/^^−^*_G12; Fig. 1A). KO of *Lrrc8a* or *Lrrc8d* did not affect cell growth overall (Fig. 1B). When we treated these *Lrrc8a^−^^/^^−^* and *Lrrc8d^−^^/^^−^* cells with cisplatin (Fig. 1C and D) and carboplatin (Fig. 1E and F), we observed an increased survival using clonogenic assays, the *Lrrc8a^−^^/^^−^* cells being more resistant than the *Lrrc8d^−^^/^^−^*cells. In contrast, only a minor effect was observed in response to oxaliplatin in the LRRC8D-deficient cells (Fig. 1G and H). Furthermore, we observed resistance of the KO cell lines to the protein synthesis inhibitor blasticidin S (Supplementary Fig. S2A and S2B). The uptake of this drug is known to be LRRC8D dependent (39). The reintroduction of the *Lrrc8a* cDNA (Supplementary Fig. S2C–E) resensitized the *Lrrc8a^−^^/^^−^* cells to both cisplatin and carboplatin as well as to blasticidin S in both polyclonal and monoclonal rescue lines, whereas no clear effect was detected for oxaliplatin (Fig. 1I and J; Supplementary Fig. S2F–J). For the *Lrrc8d^−^^/^^−^* cell lines, the reconstitution of the *Lrrc8d* cDNA also resensitized cells to cisplatin, carboplatin, and blasticidin S (Fig. 1K and M; Supplementary Fig. S2H–J), with only a minor effect for oxaliplatin (Supplementary Fig. S2I and S2J).

We further corroborated these data using polyclonal cells that we generated with single *Lrrc8a*- or *Lrrc8d*-targeting sgRNAs (Supplementary Fig. S3). With the help of the TIDE analysis (28), we quantified the presence of WT and *Lrrc8a/d*-modified alleles in the polyclonal cell populations (Supplementary Fig. S3A and S3B). These polyclonal cell populations, that contain about 50% WT alleles, were then treated with the Pt-based agents cisplatin, carboplatin, and oxaliplatin (Supplementary Fig. S3C). As expected by the presence of WT alleles, the resistance to cisplatin and carboplatin was milder compared with the KO clones presented in Fig. 1 (Supplementary Fig. S3D). More importantly, when we measured the frequency of frameshift modifications following drug treatment, we observed a clear selection in favor of the *Lrrc8a-* and Lrrc8*d*-mutated alleles following cisplatin and carboplatin. Regarding oxaliplatin, only for the *Lrrc8d*-mutated alleles, we observed a modest positive selection when treating with the 0.5 μmol/L drug concentration (Supplementary Fig. S3E and S3F).

**FIGURE 2:**
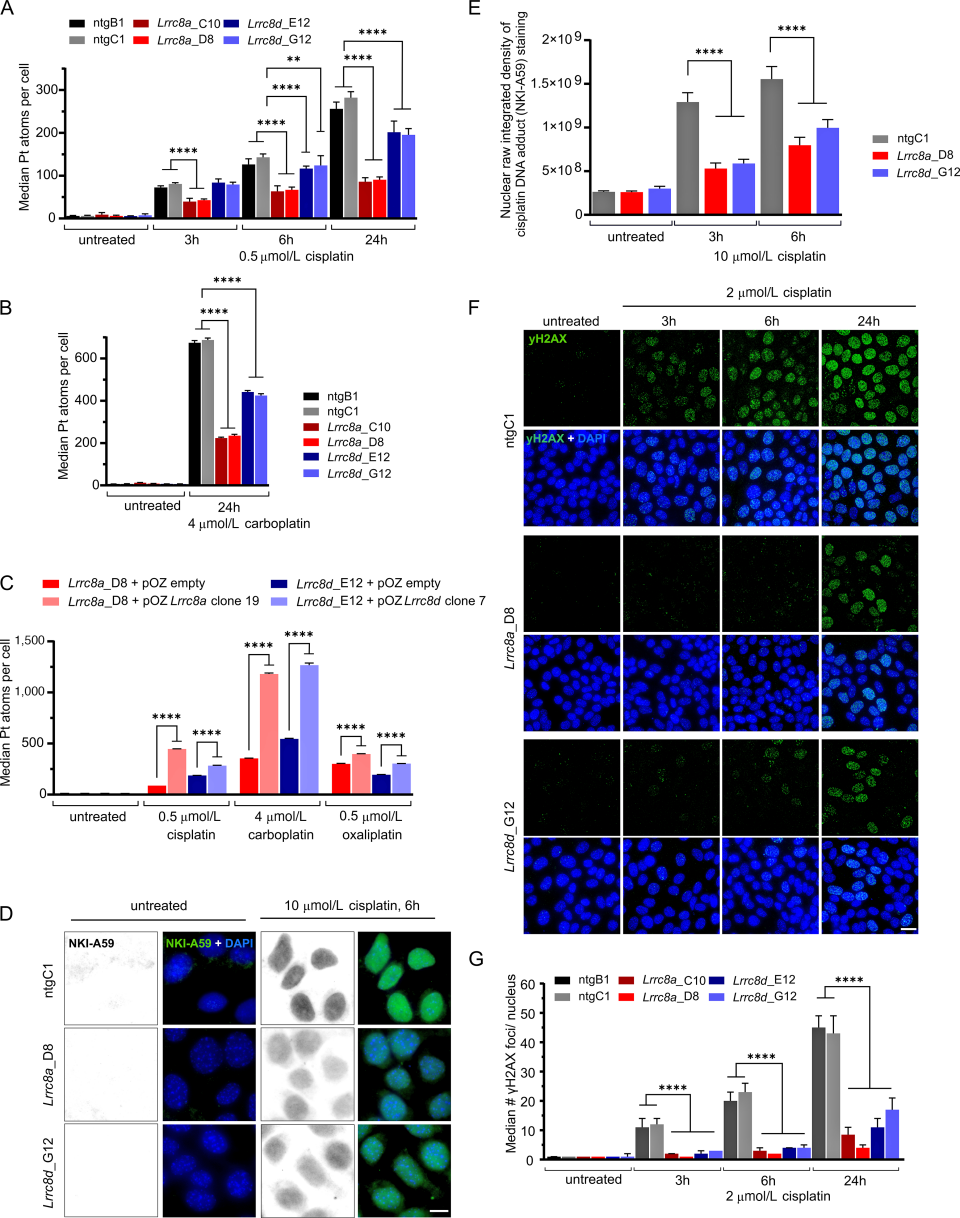
Loss of *Lrrc8a* or *Lrrc8d* reduces cisplatin uptake and the subsequent formation of Pt-DNA adducts and DNA damage. **A,** CyTOF-based measurement of Pt uptake over time using 0.5 μmol/L cisplatin in nontargeting (ntg) control, *Lrrc8a-,* or *Lrrc8d*-KO cell lines. The data represent the mean ± SD of three independent replicates consisting of three technical replicates each, where approximately 100,000 cells per condition and cell lines were acquired (two-way ANOVA followed by Tukey multiple comparisons test, ****, *P* < 0.0001; **, *P* < 0.01). **B,** CyTOF-based measurement of Pt uptake after 24 hours treatment with 4 μmol/L carboplatin of ntg, *Lrrc8a-,* or *Lrrc8d*-KO cell lines. The data represent the mean ± SD of three independent replicates where approximately 50,000 cells per condition and cell lines were acquired (two-way ANOVA followed by Tukey multiple comparisons test, ****, *P* < 0.0001). **C,** CyTOF-based measurement of Pt uptake after 24 hours treatment with 0.5 μmol/L cisplatin, 4 μmol/L carboplatin, or 0.5 μmol/L oxaliplatin of selected clonal *Lrrc8a-,* or *Lrrc8d*-high expression rescue lines compared with the empty vector transduced KO cell line. The data represent the mean ± SD of three independent replicates where approximately 50,000 cells per condition and cell lines were acquired (two-way ANOVA followed by Tukey multiple comparisons test, ****, *P* < 0.0001). **D,** Representative images of the average nuclear staining in ntg or *Lrrc8a-* or *Lrrc8d*-KO cell lines using the NKI-A59 antibody against cisplatin-adducts in the presence or absence of 10 μmol/L cisplatin treatment for 6 hours; scale bar, 10 μm. **E,** Quantification of the raw integrated density per nucleus of the NKI-A59 cisplatin adduct staining, mean with ± 95% confidence interval of three independent replicates are shown. Per replicate approximately 100 nuclei were quantified. The significance was determined using two-way ANOVA followed by Tukey multiple comparison test. ****, *P* < 0.0001. **F,** Representative images of yH2AX immunofluorescence staining of *Lrrc8a^−^^/^^−^*, *Lrrc8d^−^^/^^−^*, and control cell lines following cisplatin treatment. Scale bar, 20 μm. **G,** Quantification of yH2AX foci in the nucleus of *Lrrc8a^−^^/^^−^*, *Lrrc8d^−^^/^^−^* and control cell lines in response to cisplatin treatment. Per cell line and condition, 200 nuclei were quantified each replicate. Median ± 95% confidence interval of three independent replicates are shown (ordinary one-way ANOVA followed by Tukey multiple comparisons test, ****, *P* < 0.0001).

In our previous study using human HAP1 cells, we found that less carboplatin enters the cells if they contained VRACs that were LRRC8A or LRRC8D deficient (21). We also measured the Pt uptake in our *Lrrc8a^−^^/^^−^* and *Lrrc8d^−^^/^^−^* KB1PM5 cells using CyTOF, which allows the use of more physiologic drug concentrations than were tested previously in the HAP1 cells. After incubation with 0.5 μmol/L of cisplatin or 4 μmol/L carboplatin for 24 hours, the Pt content was reduced by more than 65% in the *Lrrc8a^−^^/^^−^* cells for both cisplatin and carboplatin and about 25% for cisplatin and 35% for carboplatin in the *Lrrc8d^−^^/^^−^* cells (Fig. 2A and B). The decrease could be reversed by the reintroduction of *Lrrc8a* or *Lrrc8d* (Fig. 2C). The decrease in intracellular Pt accumulation can be expected to result in less Pt-DNA adducts. To test this, we used the NKI-A59 antibody (31), which detects cisplatin-induced DNA adducts. Consistent with the uptake data, less Pt-DNA adducts were formed in the LRRC8A- and LRRC8D-deficient cells (Fig. 2D and E). Again, the effect was stronger in the *Lrrc8a^−^^/^^−^* cells than the *Lrrc8d^−^^/^^−^* cells. The lower amount of Pt-DNA adducts resulted in less DNA damage, as measured by γH2AX foci formation (Fig. 2F and G). Hence, the high cisplatin sensitivity of the BRCA1-deficient cells is alleviated in the absence of LRRC8A or LRRC8D, due to reduced drug uptake.

### LRRC8A and LRRC8D Defects Abrogate the *In Vivo* Efficacy of Carboplatin

**FIGURE 3:**
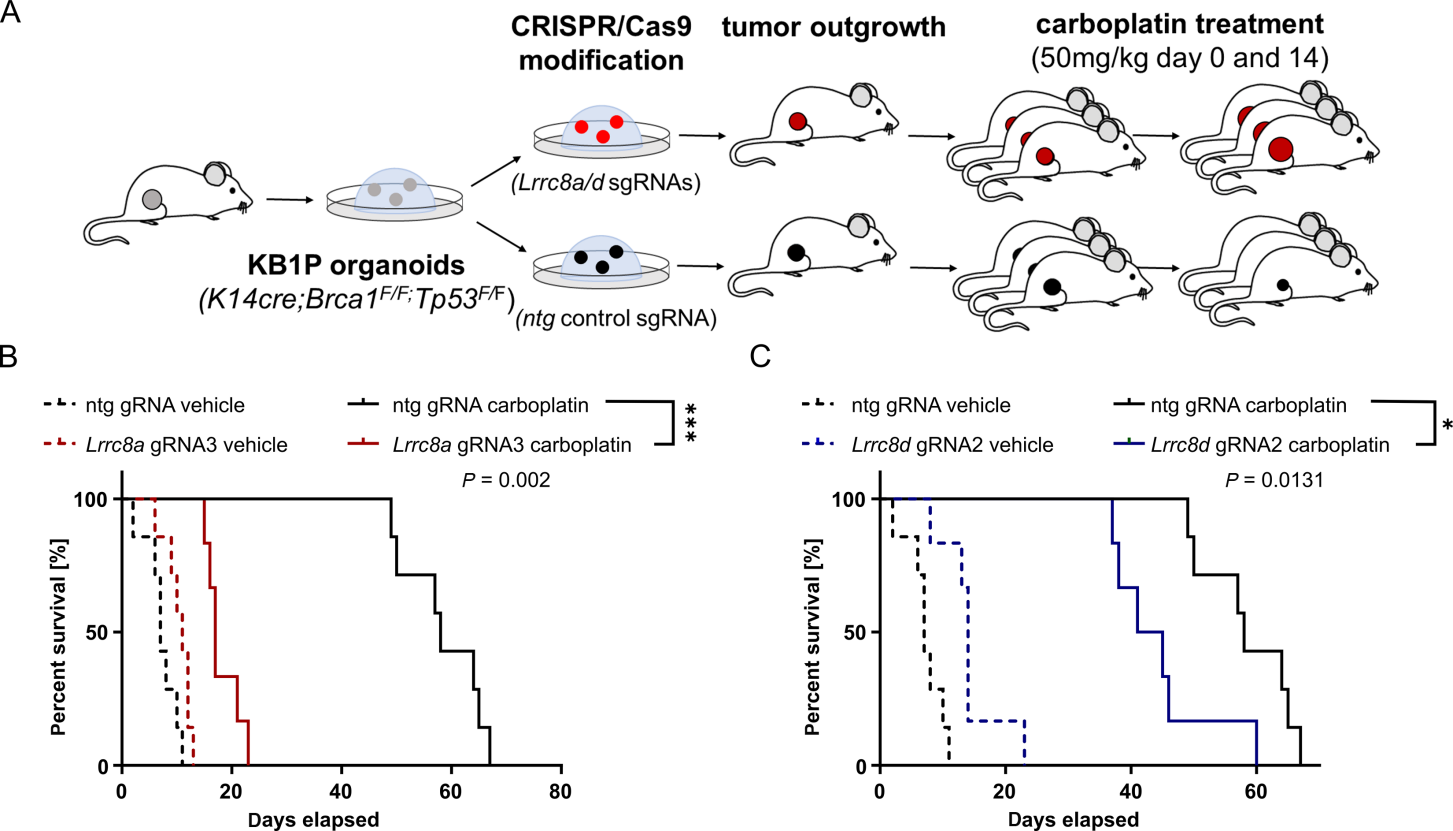
*Lrrc8a* and *Lrrc8d* deficiency promote carboplatin resistance *in vivo.***A,** Schematic overview of the *in vivo* experiment. **B** and **C,** Kaplan–Meier OS curves of mice transplanted with LRRC8A/D-deficient or ntg control tumors treated with either vehicle or 50 mg/kg carboplatin. Statistical analysis was performed with the log-rank test (Mantel–Cox). *, *P* < 0.05; ***, *P* < 0.001.

Many clinicians prefer carboplatin to cisplatin, as the toxicity profiles of the two drugs differ, including a lower nephrotoxicity of carboplatin than cisplatin (40). To validate the role of LRRC8A and LRRC8D in the Pt drug response *in vivo*, we tested the carboplatin response of the tumors in our breast cancer KB1P model, using the 3D organoid technology (26). For this purpose, KB1P4N organoids, derived from a *Brca1^−^^/^^−^;Trp53^−^^/^^−^* mammary tumor (41), were transduced with lentiviruses carrying *Lrrc8a* or *Lrrc8d* targeting pLENTiCRISPRv2 vectors. Control organoids were generated by transduction with pLentiCRISPRv2, encoding a nontargeting sgRNA. LRRC8A/D-deficient organoids were then transplanted orthotopically into the mammary fat pad of mice. The outgrowing tumors were analyzed by TIDE and indeed showed a high frameshift modification percentage (Supplementary Fig. S4A and S4B). When the organoid-derived control or Lrrc8a/d-targeted tumors reached a volume of 75 mm^3^, we treated them with the MTD of 50 mg/kg carboplatin i.v. on days 0 and 14 (Fig. 3). The tumor volume was monitored throughout the whole experiment (Supplementary Fig. S4C and S4D). As shown in Fig. 3, depletion of Lrrc8a or Lrrc8d significantly lowered the tumor response to carboplatin and resulted in a decreased overall survival (OS; *P* = 0.002 for Lrrc8a, *P* = 0.0131 for Lrrc8d; Fig. 3). Consistent with the *in vitro* data, the effect was stronger in the LRRC8A-deficient tumors than in the LRRC8D-deficient ones. These data demonstrate that loss of Lrrc8a or Lrrc8d renders BRCA1;p53-deficient tumors resistant to carboplatin *in vivo*.

### *Lrrc8d^−^^/^^−^* Mice are Viable and Provide a Useful Model to Study High-dose Cisplatin Therapy

**FIGURE 4:**
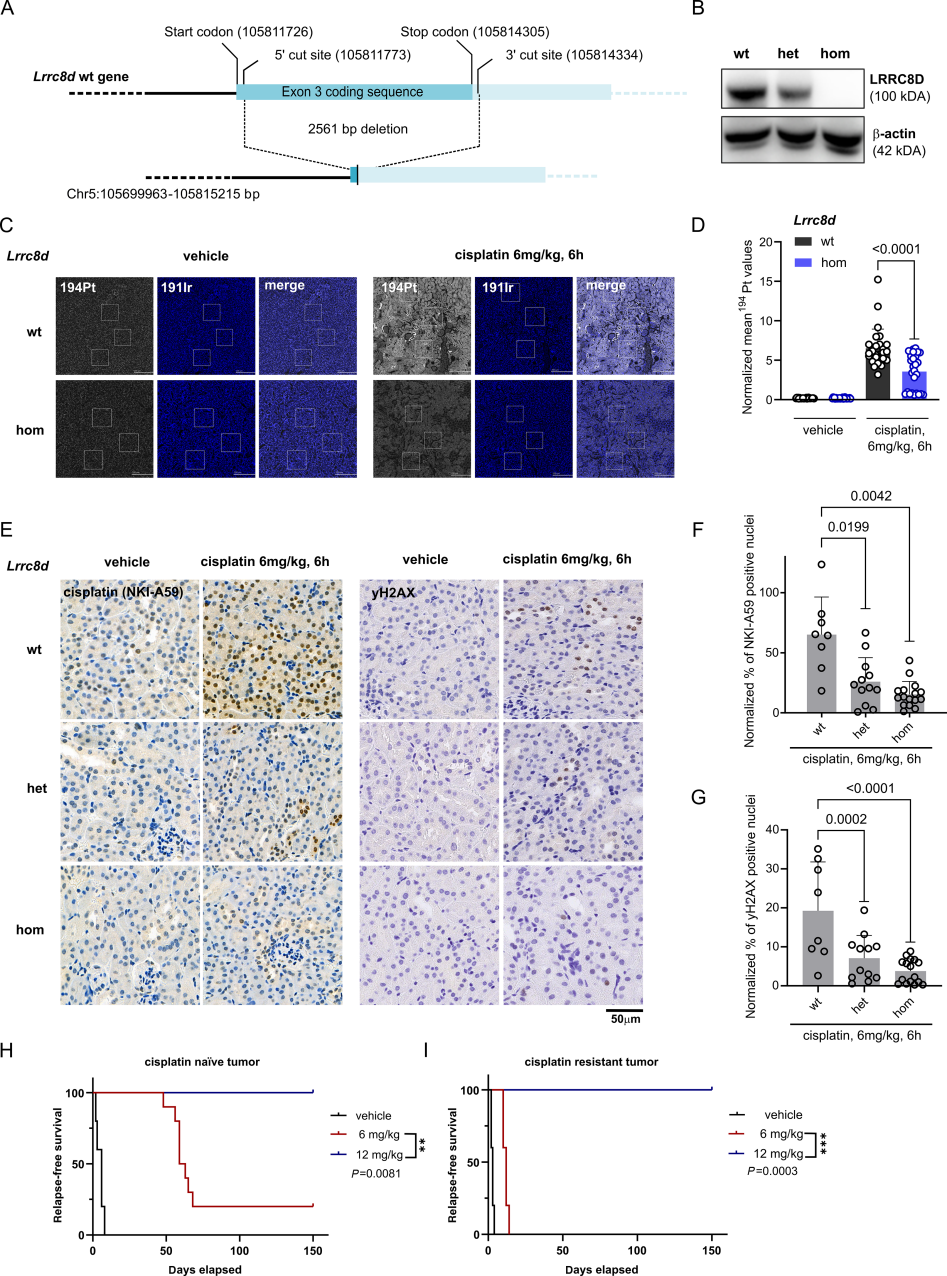
Cisplatin uptake into the kidneys of LRRC8D-deficient mice and subsequent DNA damage are reduced. **A,** A schematic overview of the development of *Lrrc8d* KO mice using CRISPR/Cas9-mediated KO of the first coding exon (2561 bp) in zygotes. **B,** Western blotting of LRRC8D using kidney lysates derived from WT, heterozygous and homozygous *Lrrc8d* KO mice. **C,** Representative images of the IMC tissue analysis of cisplatin- or vehicle-treated WT or LRRC8D-deficient mice. For the illustration, the most abundant isotope ^194^Pt was used. For the visualization of the nuclei within the tissue, the signal of the iridium DNA intercalator isotope ^191^Ir is shown. Highlighted are three image sections which were used for the quantification of the mean Pt levels. Scale bar, 200 μm. **D,** Normalized mean ^194^Pt kidney measurements of WT or LRRC8D deficient mice, 6 hours after treatment with 6 mg/kg cisplatin i.v. or the vehicle. Three 1 mm^2^ sections of three kidneys per group were acquired, where three image sections from the cortical region of each acquisition were quantified. This results in 27 datapoints per group. The data represent the ^134^Xe^+^ normalized mean ^194^Pt signal of the image sections ± SD (two-way ANOVA, followed by Tukey multiple comparisons test). **E,** IHC using the anti-cisplatin-DNA adduct and anti-yH2AX antibodies of WT, heterozygous and homozygous *Lrrc8d* KO mice after the treatment with 6 mg cisplatin per kg for 6 hours. The images were taken at 40× magnification. Scale bar, 50 μm. **F** and **G,** The percentage of positive nuclei in 50 kidney cortex image sections per kidney (for NKI-A59 antibody) or the whole cortical kidney region of each mouse (for yH2AX) were quantified and normalized via the average basal levels of the untreated samples; vehicle: wt *N* = 5, het KO *N* = 8, hom KO *N* = 8, treated: wt *N* = 8, het KO *N* = 12, hom KO *N* = 16; ****, *P* < 0.0001. The data represent mean percentage of positive nuclei over all the image sections per mouse ± SD (two-way ANOVA, followed by Tukey multiple comparisons test). Kaplan–Meier relapse-free survival of mice transplanted with either cisplatin-naïve tumors (**H**) or cisplatin resistant tumors (**I**). The mice were treated on days 0 and 14 with the indicated dose of cisplatin (i.v.). For the treatment with 12 mg/kg i.v. the *Lrrc8d* KO mice were used. Statistical analysis was performed with the log-rank test (Mantel–Cox). **, *P* < 0.01; ***, *P* < 0.001.

It was previously shown that *Lrrc8a* KO mice are severely compromised and show an increased mortality in utero and postnatally (42). To test whether the *Lrrc8d* KO is tolerable in mice, we introduced a large 2561 bp deletion of the protein coding sequence in exon 3 using CRISPR/Cas9-mediated gene editing of FVB/N zygotes (Fig. 4A). This completely abrogated *Lrrc8d* expression at the RNA and protein level in homozygous mice, and also significantly reduced RNA and protein levels in heterozygous animals (Fig. 4B; Supplementary Fig. S5A and S5B). In contrast to the *Lrrc8a* KO mice, we did not see any alterations regarding fertility or histomorphology of the *Lrrc8d^−^^/^^−^* mice that we followed for at least 6 months. We therefore conclude that the *Lrrc8d^−^^/^^−^* mice are fully viable. In FVB/N mice, we previously reported an MTD of 6 mg/kg cisplatin i.v. on days 0 and 14 (38). We could not escalate the dose above MTD levels by bone marrow reconstitution, and 3 days after treatment using 9 or 12 mg/kg cisplatin i.v., the body weight dropped below 90% and animals had to be sacrificed. In contrast, in the *Lrrc8d^−^^/^^−^* mice we could double the dose to 12 mg cisplatin per kg and the average weight of the mice stayed above 90% (Supplementary Fig. S5C). A main cytotoxic side effect of cisplatin is the increased death of tubular epithelial cells in the renal cortex. We therefore directly measured the Pt content in the kidneys of the *Lrrc8d*-proficient and homozygous KO mice 6 hours after treatment with 6 mg/kg cisplatin i.v. For the analysis, the amount of Pt was measured in three kidneys per group using IMC. The mean ^194^Pt values per section were normalized to the ^134^Xe^+^ background signal intensity. Indeed, the amount of Pt that we found in the kidneys of *Lrrc8d^−^^/^^−^* mice was lowered by about 50% compared with the WT mice (Fig. 4C and D). With less cisplatin entering the kidneys, we expected a reduction in the cisplatin-DNA adducts to be formed in the nuclei of tubular epithelial cells, which cause the nephrotoxicity. As presented in Supplementary Fig. S5D and S5E, these cells can be detected with the NKI-A59 antibody against Pt-DNA adducts 6 hours after treatment of WT mice with 6 mg/kg cisplatin i.v. The amount of Pt-DNA adducts was also substantially decreased in the kidneys of *Lrrc8d^−^^/^^−^* mice and to a lesser extent also in the *Lrrc8d^+/^^−^* animals, in comparison with the WT mice (Fig. 4E and F). This decrease of Pt-DNA adducts correlated with a decrease in DNA damage, measured by γH2AX foci formation (Fig. 4E–G). These data show that also *in vivo* less cisplatin enters LRRC8D-deficient cells.

Despite the high cisplatin sensitivity of the KB1P tumors, the tumors are not easily eradicated, not even with repeated treatments using the MTD (38). We previously showed that residual G_0_-like tumor cells that transiently avoid entering the cell cycle can escape the cisplatin-induced DNA damage, even without functional BRCA1 (38). The fact that *Lrrc8d^−^^/^^−^* mice tolerate the double MTD of the WT mice allowed us to address the basic question whether a high dose of cisplatin eradicates the KB1P tumors containing functional VRACs. As shown in Fig. 4H this is indeed the case. The KB1P donor tumor transplanted orthotopically into syngeneic WT FVB/N relapsed after about 60 days following 6 mg/kg cisplatin i.v. on days 0 and 14. In contrast, no tumor relapse was detected when the same KB1P tumor was transplanted orthotopically into syngeneic *Lrrc8d^−^^/^^−^* FVB/N mice and treated with 12 mg/kg cisplatin i.v. on days 0 and 14. By applying repeated dosing of 4 mg/kg cisplatin i.p. in WT FVB/N mice (hence 2/3 of the MTD), we also managed to generate cisplatin-resistant KB1P tumors that showed stable resistance to 6 mg/kg i.v. when transplanted into FVB/N mice (Fig. 4I). Even these resistant tumors were eradicated using the high-dose therapy (12 mg/kg) in *Lrrc8d^−^^/^^−^* mice. These data show that both sensitive and resistant *Brca1;Trp53*-deficient tumors cannot compensate the damage induced by high-dose cisplatin entering the tumor cells.

### Low Expression of LRRC8A or LRRC8D Correlates with Decreased OS and Reduced Recurrence-free Survival in Patients with HNSCC Treated with Cisplatin-based Chemoradiotherapy

In the clinic, the use of Pt drugs for the treatment of patients with BRCA-deficient breast cancer has only recently been expanded, and currently the sample availability for this specific subgroup is scarce. Another tumor type in which BRCA mutations are frequently found and which is highly sensitive to Pt-based chemotherapy is ovarian cancer. Using The Cancer Genome Atlas and Patch and colleagues datasets, we have previously reported a lower survival of patients with ovarian cancer who have a low *LRRC8D* gene expression (21). We could confirm this in another independent data set of patients with ovarian cancer (GSE32063; ref. 37). As shown in Supplementary Fig. S6, patients expressing low *LRRC8D* levels had a significantly decreased OS (*P* = 0.009) and nonsignificantly shortened progression-free survival (*P* = 0.096).

**FIGURE 5:**
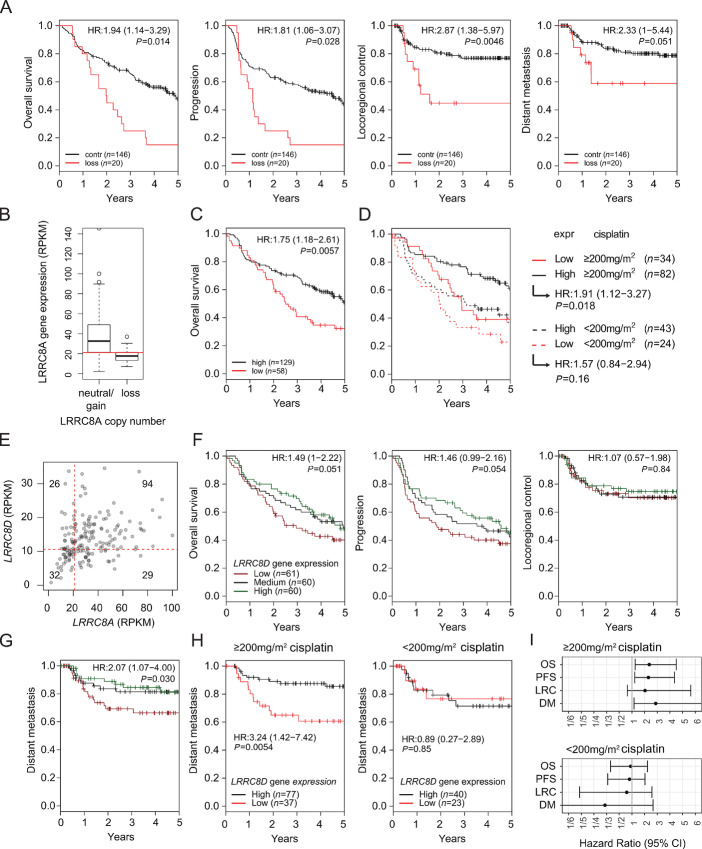
Loss of *LRRC8A* or *LRRC8D* reduces survival parameters in patients with HNSCC treated with a chemoradiotherapy regimen. **A,** OS, progression, locoregional control, and distant metastasis (DM) parameters of patients with at least −1 *LRRC8A* copy-number loss versus control. **B,** Comparison of polyA RNA-seq expression data to copy-number loss of *LRRC8A*. Patients were classified into "low" expression group by the cutoff at around 20 rpkm. **C,** OS data of high (*N* = 129) and low (*N* = 58) *LRRC8A*-expressing patients defined by the cutoff determined in **B**. **D,** Patients were further classified into received cumulative dose groups of below or above 200 mg/m^2^ of cisplatin. **E,** Correlation analysis of *LRRC8A* versus *LRRC8D* expression levels. **F,** OS, progression, and locoregional control parameters of patients differentially expressing *LRRC8D* (low *N* = 61, medium *N* = 60, high = 60). **G,** Distant metastasis outcome of patients with low, medium, or high *LRRC8D* expression. **H,** Distant metastasis outcome of patients differentially expressing *LRRC8D* further classified in high cumulative dose (left) and low cumulative dose of cisplatin (right). **I,** Forest plots displaying the results from CoxPH model fits for OS, PFS, locoregional control, and DM-free survival for the >200 mg/m^2^ of cisplatin or <200 mg/m^2^ of cisplatin treatment groups.

As the relevance of LRRC8A or LRRC8D function for Pt drug uptake is independent of BRCA1/2 status, we also studied LRRC8A/D in patients with HNSCC, for which cisplatin is a standard therapy in combination with radiotherapy (Fig. 5). In contrast to the ovarian cancer cohort, we had both copy-number variation (CNV) and gene expression data available from the tumors of these patients with HNSCC. CNV analysis identified 20 of 166 tumors with a loss of the *LRRC8A* gene. In these patients, the loss of *LRRC8A* is associated with a lower OS (*P* = 0.014) and increased tumor progression (*P* = 0.028) compared. Moreover, the loss of *LRRC8A* in these patients is correlated with an increased risk for failure of locoregional control (*P* = 0.0046) and distant metastasis (*P* = 0.051) after treatment (Fig. 5A). To test for alterations in the level of *LRRC8A* transcripts, gene expression data of these patients were analyzed. Samples were classified to low (<20 rpkm, *N* = 58) and high (>20 rpkm, *N* = 129) subgroups (Fig. 5B). Low *LRRC8A* gene expression correlated significantly with poor OS in these patients (*P* = 0.0057; Fig. 5C).

It has previously been shown that patients benefit from cumulative cisplatin-based radiotherapy regimens above 200 mg/m^2^ (43, 44). We therefore stratified patients according to their cumulative dose, and their OS was analyzed according to their *LRRC8A* expression levels (for low *LRRC8A* expression *N* = 43 in ≥200 mg/m^2^ and *N* = 24 in <200 mg/m^2^). The loss of *LRRC8A* expression resulted in a decrease of the OS curves to the levels of lower and less effective cisplatin doses (Fig. 5D). The data suggest that HNSCC patients with high *LRRC8A* expression are likely to benefit from higher cisplatin doses.

Regarding *LRRC8D*, we also identified a group of 61 patients with low gene expression, and these patients are different from those expressing low levels of *LRRC8A* (Fig. 5E). The stratification of the tumors into low (*N* = 61), medium (*N* = 60), and high (*N* = 60) *LRRC8D* gene expression subgroups revealed decreased OS (*P* = 0.051) and poor tumor progression outcome (*P* = 0.054) in the low expression group. No effects on locoregional control of the tumor were found (*P* = 0.84; Fig. 5F). Moreover, low *LRRC8D* expression correlated with an increased distant metastasis occurrence (*P* = 0.030; Fig. 5G). To assess the impact of *LRRC8D* expression on varying cumulative cisplatin doses, patients were split into ≥200 mg/m^2^ and <200 mg/m^2^ receiving subgroups. Low expression was defined as the lowest tertile expressing group in both treatment classes (*N* = 37 in ≥200 mg/m^2^ and *N* = 23 in <200 mg/m^2^). Low *LRRC8D* gene expression was associated with increased distant metastasis, especially in the ≥200 mg/m^2^ subgroup of patients (*P* = 0.0054; Fig. 5H). This association was strongest in the distant metastasis formation (Fig. 5I).

Hence, in cisplatin-treated patients with HNSCC, low *LRRC8A* and *LRRC8D* expression is associated with poor outcome. On the basis of our experimental work, this is explained by poor drug uptake of these tumors.

## Discussion

Using a mouse model for *BRCA1*-mutated breast cancer, we show here the relevance of LRRC8A- and LRRC8D-mediated cisplatin and carboplatin uptake to kill Pt drug–sensitive tumors. The potential relevance to the treatment of human cancer is suggested by our finding that patients with ovarian cancer as well as HNSCC with low gene expression levels of *LRRC8A* or *LRRC8D* in their tumors have a reduced benefit of Pt-based chemotherapy. Hence, the absence of LRRC8A or LRRC8D in tumor cells may be a helpful marker to avoid the use of cisplatin or carboplatin.

At present, there is no biomarker routinely applied in the clinic to predict the outcome of Pt-based chemotherapy. In part, this may be due to the fact that cisplatin influx into tumor cells was long thought to be completely due to passive diffusion (15). Although active influx via SLC31A1—the mammalian homolog of the budding yeast copper transporter (CTR1)—or via OCT2 (SLC22A2)—an organic cation transporter—was suggested, these transporters could not be unambiguously proven to cause reduced uptake and drug resistance when absent (45, 46). OCT2 has been shown to mediate cisplatin-induced nephrotoxicity (47, 48), but its expression is largely limited to the kidney, explaining why it may not be involved in tumor uptake. Decreased accumulation of Pt drugs may also be caused by increased drug export. In this context, two Cu transporters—ATP7A and 7B—were put forward (49). Despite some correlations that have been reported between poor cisplatin response and high ATP7B levels in patients, the role of these efflux transporters in Pt drug resistance remains to be clarified (2, 15, 20). Moreover, low-level cisplatin resistance was reported in cells overexpressing *MRP2*, but in human tumors, there is no consistent correlation between *MRP2* expression and cisplatin resistance (15, 46). In summary, no transporter has been explicitly linked to clinical Pt drug resistance, be it importer or exporter (2, 20).

In addition to diffusion or transporters, channels provide another route for Pt drugs to enter cells. Already in 1993, Gately and Howell (50) concluded that a fraction of cisplatin enters via a channel, because there are several inhibitors that decrease cisplatin entry. The authors suggest that about half of the cisplatin uptake is by passive diffusion through the membrane and the other half through a dedicated channel. More than 50% inhibition by any inhibitor has not been seen, resulting in this 50% channel estimate. Using genome-wide functional genetic screens for carboplatin drug resistance in human HAP1 cells, we have recently identified VRACs composed of LRRC8A and LRRC8D proteins as these long sought-after plasma membrane entry points for cisplatin and carboplatin (21). Loss of LRRC8D was also a major hit in a genome-scale CRISPR-Cas9 KO screen for cisplatin resistance using *BRCA1*-mutated ovarian cancer cells (51). Here, we confirm the relevance of these proteins for sensitivity to Pt drugs, using Pt drug–sensitive BRCA1;p53-deficient mouse mammary tumors and cell lines derived from this model. Despite their strong drug sensitivity due to an irreversibly deleted *Brca1* gene, the effect of cisplatin and carboplatin was largely abrogated in the absence of LRRC8A or LRRC8D. We found that about 50% of carboplatin and cisplatin uptake depended on LRRC8A and LRRC8D (Fig. 2), which is consistent with the assumption of Gately and Howell (50) and our observations in HAP1 cells (21). According to recent Cryo-EM studies of the LRRC8 subunits, the substrate specificity for larger osmolytes is likely dependent on the presence of LRRC8D in the channel composition. LRRC8D is the largest isoform, presenting the longest extracellular loop between transmembrane domain 1 and 2 (52, 53). Of note, homo-hexameric LRRC8D structures contain a wider pore diameter than structures consisting of solely LRRC8A (53, 54). Together with LRRC8A, LRRC8D seems to be responsible for the cellular uptake of Pt-based drugs, in contrast to the other subunits (LRRC8C, LRRC8E; ref. 21). However, the effect of LRRC8A depletion on Pt drug resistance is not seen in all cell lines though, most likely because LRRC8A is essential for some of them (24, 55). *Lrrc8a* KO mice are severely compromised and show an increased mortality *in utero* and postnatally, as well as infertility (42, 56). In contrast, we found that *Lrrc8d* KO mice are viable and breed normally. Although further experiments are required to investigate Pt drug pharmacokinetics, renal excretion and nephrotoxicity, we clearly observed less cisplatin and Pt-DNA adducts in the kidneys of *Lrrc8d*^−/−^ mice, resulting in reduced DNA damage. Because of reduced cisplatin uptake, their MTD is doubled, which allowed us to build a model for high-dose Pt drug–based chemotherapy. Both cisplatin-sensitive and -resistant LRRC8A- and LRRC8D-proficient KB1P tumors were completely eradicated when the cisplatin MTD was augmented 2-fold (Fig. 4). This is consistent with our previous findings using nimustine (38), that drug-tolerant cells can be eliminated, if sufficient damage is inflicted. Also in our cohort of patients with HNSCC, the correlation between low *LRRC8A* or *LRRC8D* gene expression and poor cisplatin-based therapy response was more pronounced in the high-dose groups. While further analyses need to be made to investigate whether this result can be reproduced using carboplatin, these data strongly indicate that it would be useful to assess the *LRRC8A* and *LRRC8D* expression of tumor cells before applying high-dose cisplatin- or carboplatin-based therapies. Clinicians might be able to avoid the serious side effects, if insufficient drug amounts can be expected to reach the tumor cell DNA, the main cellular target of Pt drugs. An alternative approach for cancers with low LRRC8A levels may be oxaliplatin. In our KB1P model, we only observed a modest reduction in oxaliplatin uptake and consequent therapy resistance in *Lrrc8d*-deficient cells, suggesting oxaliplatin as useful alternative when patients are stratified on the basis of *LRRC8A* expression.

Given the frequent use of Pt drugs in daily clinical practice, our data highlight the importance of further validation of LRRC8A and LRRC8D status as a predictive biomarker in prospective clinical trials. In addition to classical gene or protein expression correlations, our results also suggest the use of CyTOF-based measurements to quantitatively evaluate Pt uptake in patient-derived primary 2D or 3D cell cultures.

## Supplementary Material

Table TS1 gRNA oligos used for the generation of Lrrc8a or Lrrc8d-knockout cell lines, organoids and tumors

Table TS2 Primer sequences used for Lrrc8a or Lrrc8d knockout control in 2D cell lines or Lrrc8d knockout mice or reconstitution experiments

Figure FS1 Generation of monoclonal knockout cell lines of Lrrc8a or Lrrc8d used in main Figure

Figure FS2 Blasticidin and Pt treatment of Lrrc8a or Lrrc8d rescue cell lines data associated with main Figure 1

Figure FS3 Pt-drug treatment of polyclonal cell lines with wild type as well as Lrrc8a or Lrrc8d knockout alleles shows positive selection for knockout alleles

Figure FS4 TIDE analysis and tumor growth curves of Kaplan Meyer survival graphs shown in main Figure 3

Figure FS5 Lrrc8d KO mice tolerate higher cisplatin doses than wild type mice

Figure FS6 Analysis of publicly available ovarian cancer dataset to identify the association of LRRC8A or LRRC8D expression with outcome of chemotherapy with cisplatin

## References

[1] Kelland L . The resurgence of platinum-based cancer chemotherapy. Nat Rev Cancer 2007;7:573–84. 17625587 10.1038/nrc2167

[2] Rottenberg S , Disler C, Perego P. The rediscovery of platinum-based cancer therapy. Nat Rev Cancer 2021;21:37–50. 33128031 10.1038/s41568-020-00308-y

[3] Pujade-Lauraine E , Fujiwara K, Ledermann JA, Oza AM, Kristeleit R, Ray-Coquard IL, . Avelumab alone or in combination with chemotherapy versus chemotherapy alone in platinum-resistant or platinum-refractory ovarian cancer (JAVELIN Ovarian 200): an open-label, three-arm, randomised, phase 3 study. Lancet Oncol 2021;22:1034–46. 34143970 10.1016/S1470-2045(21)00216-3

[4] Burtness B , Harrington KJ, Greil R, Soulières D, Tahara M, de Castro G, . Pembrolizumab alone or with chemotherapy versus cetuximab with chemotherapy for recurrent or metastatic squamous cell carcinoma of the head and neck (KEYNOTE-048): a randomised, open-label, phase 3 study. Lancet 2019;394:1915–28. 31679945 10.1016/S0140-6736(19)32591-7

[5] Gandhi L , Rodríguez-Abreu D, Gadgeel S, Esteban E, Felip E, De Angelis F, . Pembrolizumab plus chemotherapy in metastatic non-small-cell lung cancer. N Engl J Med 2018;378:2078–92. 29658856 10.1056/NEJMoa1801005

[6] Lippard SJ . New chemistry of an old molecule: cis-[Pt(NH3) 2Cl2]. Science 1982;218:1075–82. 6890712 10.1126/science.6890712

[7] Lord CJ , Ashworth A. The DNA damage response and cancer therapy. Nature 2012;481:287–94. 22258607 10.1038/nature10760

[8] Melinda LT , Kirsten MT, Julia R, Bryan H, Gordon BM, Kristin CJ, . Homologous recombination deficiency (hrd) score predicts response to platinum-containing neoadjuvant chemotherapy in patients with triple-negative breast cancer. Clin Cancer Res 2016;22:3764–73. 26957554 10.1158/1078-0432.CCR-15-2477 PMC6773427

[9] Vollebergh MA , Lips EH, Nederlof PM, Wessels LFAA, Schmidt MK, van Beers EH, . An aCGH classifier derived from BRCA1-mutated breast cancer and benefit of high-dose platinum-based chemotherapy in HER2-negative breast cancer patients. Ann Oncol 2011;22:1561–70. 21135055 10.1093/annonc/mdq624 PMC3121967

[10] Vollebergh MA , Lips EH, Nederlof PM, Wessels LFAA, Wesseling J, Vd Vijver MJ, . Genomic patterns resembling BRCA1- and BRCA2-mutated breast cancers predict benefit of intensified carboplatin-based chemotherapy. Breast Cancer Res 2014;16:R47. 24887359 10.1186/bcr3655 PMC4076636

[11] Silver DP , Richardson AL, Eklund AC, Wang ZC, Szallasi Z, Li Q, . Efficacy of neoadjuvant cisplatin in triple-negative breast cancer. J Clin Oncol 2010;28:1145–53. 20100965 10.1200/JCO.2009.22.4725 PMC2834466

[12] Rottenberg S , Nygren AOHH, Pajic M, van Leeuwen FWBB, van der Heijden I, van de Wetering K, . Selective induction of chemotherapy resistance of mammary tumors in a conditional mouse model for hereditary breast cancer. Proc Natl Acad Sci U S A 2007;104:12117–22. 17626183 10.1073/pnas.0702955104 PMC1914039

[13] Rottenberg S , Jaspers JE, Kersbergen A, Van Der Burg E, Nygren AOHH, Zander SALL, . High sensitivity of BRCA1-deficient mammary tumors to the PARP inhibitor AZD2281 alone and in combination with platinum drugs. Proc Natl Acad Sci U S A 2008;105:17079–84. 18971340 10.1073/pnas.0806092105 PMC2579381

[14] Keener AB . Innovative therapies to tackle platinum-resistant ovarian cancer. Nature 2021;600:S45–7.

[15] Borst P , Rottenberg S, Jonkers J. How do real tumors become resistant to cisplatin? Cell Cycle 2008;7:1353–9. 18418074 10.4161/cc.7.10.5930

[16] Borst P . Cancer drug pan-resistance: pumps, cancer stem cells, quiescence, epithelial to mesenchymal transition, blocked cell death pathways, persisters or what? Open Biol 2012;2:120066. 22724067 10.1098/rsob.120066 PMC3376736

[17] Edwards SL , Brough R, Lord CJ, Natrajan R, Vatcheva R, Levine DA, . Resistance to therapy caused by intragenic deletion in BRCA2. Nature 2008;451:1111–5. 18264088 10.1038/nature06548

[18] Sakai W , Swisher EM, Karlan BY, Agarwal MK, Higgins J, Friedman C, . Secondary mutations as a mechanism of cisplatin resistance in BRCA2-mutated cancers. Nature 2008;451:1116–20. 18264087 10.1038/nature06633 PMC2577037

[19] Patch AM , Christie EL, Etemadmoghadam D, Garsed DW, George J, Fereday S, . Whole-genome characterization of chemoresistant ovarian cancer. Nature 2015;521:489–94. 26017449 10.1038/nature14410

[20] Burger H , Loos WJ, Eechoute K, Verweij J, Mathijssen RHJ, Wiemer EAC. Drug transporters of platinum-based anticancer agents and their clinical significance. Drug Resist Updat 2011;14:22–34. 21251871 10.1016/j.drup.2010.12.002

[21] Planells-Cases R , Lutter D, Guyader C, Gerhards NM, Ullrich F, Elger DA, . Subunit composition of VRAC channels determines substrate specificity and cellular resistance to Pt-based anti-cancer drugs. EMBO J 2015;34:2993–3008. 26530471 10.15252/embj.201592409 PMC4687416

[22] Qiu Z , Dubin AE, Mathur J, Tu B, Reddy K, Miraglia LJ, . SWELL1, a plasma membrane protein, is an essential component of volume-regulated anion channel. Cell 2014;157:447–58. 24725410 10.1016/j.cell.2014.03.024 PMC4023864

[23] Trothe J , Ritzmann D, Lang V, Scholz P, Pul Ü, Kaufmann R, . Hypotonic stress response of human keratinocytes involves LRRC8A as component of volume-regulated anion channels. Exp Dermatol 2018;27:1352–60. 30252954 10.1111/exd.13789

[24] Voss FK , Ullrich F, Münch J, Lazarow K, Lutte D, Mah N, . Identification of LRRC8 heteromers as an essential component of the volume-regulated anion channel VRAC. Science 2014;344:634–8. 24790029 10.1126/science.1252826

[25] Jaspers JE , Kersbergen A, Boon U, Sol W, van Deemter L, Zander SA, . Loss of 53BP1 causes PARP inhibitor resistance in BRCA1-mutated mouse mammary tumors. Cancer Discov 2013;3:68–81. 23103855 10.1158/2159-8290.CD-12-0049 PMC7518105

[26] Duarte AA , Gogola E, Sachs N, Barazas M, Annunziato S, de Ruiter JR, . BRCA-deficient mouse mammary tumor organoids to study cancer-drug resistance. Nat Methods 2018;15:134–40. 29256493 10.1038/nmeth.4535

[27] Harmsen T , Klaasen S, Van De Vrugt H, Riele HT. DNA mismatch repair and oligonucleotide end-protection promote base-pair substitution distal from a CRISPR/Cas9-induced DNA break. Nucleic Acids Res 2018;46:2945–55. 29447381 10.1093/nar/gky076 PMC5888797

[28] Brinkman EK , Chen T, Amendola M, Van Steensel B. Easy quantitative assessment of genome editing by sequence trace decomposition. Nucleic Acids Res 2014;42:e168. 25300484 10.1093/nar/gku936 PMC4267669

[29] Guzmán C , Bagga M, Kaur A, Westermarck J, Abankwa D ColonyArea: an imagej plugin to automatically quantify colony formation in clonogenic assays. PLoS One 2014;9:e92444. 24647355 10.1371/journal.pone.0092444 PMC3960247

[30] Pritchard CEJ , Kroese LJ, Huijbers IJ. Direct generation of conditional alleles using CRISPR/Cas9 in mouse zygotes. Methods Mol Biol 2017;1642:21–35. 28815491 10.1007/978-1-4939-7169-5_2

[31] Terheggen PMAB , Floot BGJ, Lempers ELM, Van Tellingen O, Begg AC, Engelsel LD. Antibodies against eisplatin-modified DNA and cisplatin-modified (di)nueleotides. Cancer Chemother Pharmacol 1991;28:185–91. 1855275 10.1007/BF00685507

[32] Giesen C , Wang HAO, Schapiro D, Zivanovic N, Jacobs A, Hattendorf B, . Highly multiplexed imaging of tumor tissues with subcellular resolution by mass cytometry. Nat Methods 2014;11:417–22. 24584193 10.1038/nmeth.2869

[33] Chang Q , Ornatsky OI, Siddiqui I, Straus R, Baranov VI, Hedley DW. Biodistribution of cisplatin revealed by imaging mass cytometry identifies extensive collagen binding in tumor and normal tissues. Sci Rep 2016;6:36641. 27812005 10.1038/srep36641 PMC5095658

[34] Schindelin J , Arganda-Carreras I, Frise E, Kaynig V, Longair M, Pietzsch T, . Fiji: an open-source platform for biological-image analysis. Nat Methods 2012;9:676–82. 22743772 10.1038/nmeth.2019 PMC3855844

[35] Essers PBM , van der Heijden M, Vossen D, de Roest RH, Leemans CR, Brakenhoff RH, . Ovarian cancer-derived copy number alterations signatures are prognostic in chemoradiotherapy-treated head and neck squamous cell carcinoma. Int J Cancer 2020;147:1732–9. 32167160 10.1002/ijc.32962 PMC7496441

[36] van der Heijden M , Essers PBM, Verhagen CVM, Willems SM, Sanders J, de Roest RH, . Epithelial-to-mesenchymal transition is a prognostic marker for patient outcome in advanced stage HNSCC patients treated with chemoradiotherapy. Radiother Oncol 2020;147:186–94. 32413532 10.1016/j.radonc.2020.05.013

[37] Yoshihara K , Tsunoda T, Shigemizu D, Fujiwara H, Hatae M, Fujiwara H, . High-risk ovarian cancer based on 126-gene expression signature is uniquely characterized by downregulation of antigen presentation pathway. Clin Cancer Res 2012;18:1374–85. 22241791 10.1158/1078-0432.CCR-11-2725

[38] Pajic M , Blatter S, Guyader C, Gonggrijp M, Kersbergen A, Küçükosmanoğlu A, . Selected alkylating agents can overcome drug tolerance of G_0_-like tumor cells and eradicate BRCA1-deficient mammary tumors in mice. Clin Cancer Res 2017;23:7020–33. 28821557 10.1158/1078-0432.CCR-17-1279

[39] Lee CC , Freinkman E, Sabatini DM, Ploegh HL. The protein synthesis inhibitor blasticidin s enters mammalian cells via Leucine-rich repeat-containing protein 8D. J Biol Chem 2014;289:17124–31. 24782309 10.1074/jbc.M114.571257 PMC4059153

[40] Crona DJ , Faso A, Nishijima TF, McGraw KA, Galsky MD, Milowsky MI. A systematic review of strategies to prevent cisplatin-induced nephrotoxicity. Oncologist 2017;22:609–19. 28438887 10.1634/theoncologist.2016-0319 PMC5423518

[41] Liu X , Holstege H, van der Gulden H, Treur-Mulder M, Zevenhoven J, Velds A, . Somatic loss of BRCA1 and p53 in mice induces mammary tumors with features of human BRCA1-mutated basal-like breast cancer. Proc Natl Acad Sci U S A 2007;104:12111–6. 17626182 10.1073/pnas.0702969104 PMC1924557

[42] Kumar L , Chou J, Yee CSK, Borzutzky A, Vollmann EH, von Andrian UH, . Leucine-rich repeat containing 8A (LRRC8A) is essential for T lymphocyte development and function. J Exp Med 2014;211:929–42. 24752297 10.1084/jem.20131379 PMC4010910

[43] Strojan P , Vermorken JB, Beitler JJ, Saba NF, Haigentz M, Bossi P, . Cumulative cisplatin dose in concurrent chemoradiotherapy for head and neck cancer: a systematic review. Head Neck 2016;38:E2151–8. 25735803 10.1002/hed.24026

[44] Al-Mamgani A , de Ridder M, Navran A, Klop WM, de Boer JP, Tesselaar ME. The impact of cumulative dose of cisplatin on outcome of patients with head and neck squamous cell carcinoma. Eur Arch Otorhinolaryngol 2017;274:3757–65. 28755023 10.1007/s00405-017-4687-4

[45] Safaei R , Howell SB. Copper transporters regulate the cellular pharmacology and sensitivity to Pt drugs. Crit Rev Oncol Hematol 2005;53:13–23. 15607932 10.1016/j.critrevonc.2004.09.007

[46] Hall MD , Okabe M, Shen DW, Liang XJ, Gottesman MM. The role of cellular accumulation in determining sensitivity to platinum-based chemotherapy. Annu Rev Pharmacol Toxicol 2008;48:495–535. 17937596 10.1146/annurev.pharmtox.48.080907.180426

[47] Filipski KK , Mathijssen RH, Mikkelsen TS, Schinkel AH, Sparreboom A. Contribution of organic cation transporter 2 (OCT2) to cisplatin-induced nephrotoxicity. Clin Pharmacol Ther 2009;86:396–402. 19625999 10.1038/clpt.2009.139 PMC2746866

[48] Ciarimboli G , Deuster D, Knief A, Sperling M, Holtkamp M, Edemir B, . Organic cation transporter 2 mediates cisplatin-induced oto- and nephrotoxicity and is a target for protective interventions. Am J Pathol 2010;176:1169–80. 20110413 10.2353/ajpath.2010.090610 PMC2832140

[49] Komatsu M , Sumizawa T, Mutoh M, Chen ZS, Terada K, Furukawa T, . Copper-transporting P-type adenosine triphosphatase (ATP7B) is associated with cisplatin resistance. Cancer Res 2000;60:1312–6. 10728692

[50] Gately DP , Howell SB. Cellular accumulation of the anticancer agent cisplatin: a review. Br J Cancer 1993;67:1171–6. 8512802 10.1038/bjc.1993.221 PMC1968522

[51] He YJ , Meghani K, Caron M-CC, Yang C, Ronato DA, Bian J, . DYNLL1 binds to MRE11 to limit DNA end resection in BRCA1-deficient cells. Nature 2018;563:522–6. 30464262 10.1038/s41586-018-0670-5 PMC7155769

[52] Abascal F , Zardoya R. LRRC8 proteins share a common ancestor with pannexins, and may form hexameric channels involved in cell-cell communication. Bioessays 2012;34:551–60. 22532330 10.1002/bies.201100173

[53] Nakamura R , Numata T, Kasuya G, Yokoyama T, Nishizawa T, Kusakizako T, . Cryo-EM structure of the volume-regulated anion channel LRRC8D isoform identifies features important for substrate permeation. Commun Biol 2020;3:240. 32415200 10.1038/s42003-020-0951-z PMC7229184

[54] Kasuya G , Nakane T, Yokoyama T, Jia Y, Inoue M, Watanabe K, . Cryo-EM structures of the human volume-regulated anion channel LRRC8. Nat Struct Mol Biol 2018;25:797–804. 30127360 10.1038/s41594-018-0109-6

[55] Ruprecht N , Hofmann L, Hungerbühler MN, Kempf C, Heverhagen JT, von Tengg-Kobligk H. Generation of stable cisPt resistant lung adenocarcinoma cells. Pharmaceuticals 2020;13:109. 32485798 10.3390/ph13060109 PMC7345436

[56] Lück JC , Puchkov D, Ullrich F, Jentsch TJ. LRRC8/VRAC anion channels are required for late stages of spermatid development in mice. J Biol Chem 2018;293:11796–808. 29880644 10.1074/jbc.RA118.003853 PMC6066314

