# Supplementary material for: Loss of the Volume-regulated Anion Channel Components LRRC8A and LRRC8D Limits Platinum Drug Efficacy
Source: Cancer Res Commun. 2022 Oct 26;2(10):1266–81. doi: 10.1158/2767-9764.CRC-22-0208 (PMC7613873; doi:10.1158/2767-9764.CRC-22-0208)
Supplement: Table TS1 — gRNA oligos used for the generation of Lrrc8a or Lrrc8d-knockout cell lines, organoids and tumors [file crc-22-0208-s01.docx]

**Table S1**

**Supplementary table 1 gRNA oligos used for the generation of *Lrrc8a* or *Lrrc8d*-knockout cell lines, organoids and tumors.** Restriction cloning overhangs are highlighted in blue; guanines that were added for U6 promotor compatibility of the gRNAs are highlighted in red.

| **Non-targeting (ntg)** | **Sequence (5'-->3')** | **Used for** |
| --- | --- | --- |
| gRNA ntg – FW oligo | CACCGTGATTGGGGGTCGTTCGCCA | Generation of control cell lines |
| gRNA ntg – RV oligo | AAACTGGCGAACGACCCCCAATCAC |  |
| ***Lrrc8a* targeting** | **Sequence (5'-->3')** | **Used for** |
| gRNA1 – FW oligo | CACCGGCTTCAGGATCCGGTATGC | Paired gRNA knockout for monoclonal cell lines with big deletion |
| gRNA1 – RV oligo | AAACGCATACCGGATCCTGAAGCC |  |
| gRNA3 – FW oligo | CACCGCCCCGGAAGGAGTCGTTGC | Competition assay in 2D lines, sgRNA knockout in organoids |
| gRNA3 – RV oligo | AAACGCAACGACTCCTTCCGGGGC |  |
| gRNA4 – FW oligo | CACCGCTCGTTCGAGTCGATCCGAG | Paired gRNA knockout for monoclonal cell lines with big deletion |
| gRNA4 – RV oligo | AAACCTCGGATCGACTCGAACGAGC |  |
| ***Lrrc8d* targeting** | **Sequence (5'-->3')** | **Used for** |
| gRNA2 – FW oligo | CACCGGACGTGTTTATGGATTACC | Competition assay in 2D lines, paired gRNA knockout for monoclonal cell lines with big deletion |
| gRNA2 – RV oligo | AAACGGTAATCCATAAACACGTCC |  |
| gRNA3 – FW oligo | CACCGTTTGCAGGCGACGGCAACAC | Paired gRNA knockout for monoclonal cell lines with big deletion, sgRNA knockout in organoids |
| gRNA3 – RV oligo | AAACGTGTTGCCGTCGCCTGCAAAC |  |
| gRNA4 – FW oligo | CACCGCGAACACCTCGTACCCCGTG | Paired gRNA knockout for monoclonal cell lines with big deletion |
| gRNA4 – RV oligo | AAACCACGGGGTACGAGGTGTTCGC |  |
| *Lrrc8d* knockout mice gRNA FW oligo1 | CACCGGCTTCAGGATTCGGTAAGT | Generation of conditional *Lrrc8d* knockout mice |
| *Lrrc8d* knockout mice gRNA FW oligo 2 | CACCGCAGGCACACCCACGTGCGG |  |
