# Supplementary material for: Loss of the Volume-regulated Anion Channel Components LRRC8A and LRRC8D Limits Platinum Drug Efficacy
Source: Cancer Res Commun. 2022 Oct 26;2(10):1266–81. doi: 10.1158/2767-9764.CRC-22-0208 (PMC7613873; doi:10.1158/2767-9764.CRC-22-0208)
Supplement: Table TS2 — Primer sequences used for Lrrc8a or Lrrc8d knockout control in 2D cell lines or Lrrc8d knockout mice or reconstitution experiments [file crc-22-0208-s02.docx]

**Table S2**

**Supplementary table 2 Primer sequences used for *Lrrc8a* or *Lrrc8d* knockout control in 2D cell lines or *Lrrc8d* knockout mice or reconstitution experiments.**

| **Primer name/used for** | **Sequence (5'-->3')** |
| --- | --- |
| Amplicon primer *Lrrc8a* FW | ACAGAGCTCCGCTACTTTGC |
| Amplicon primer *Lrrc8a* RV | GGATGGTCACGTCGGGTATC |
| Amplicon primer *Lrrc8d* FW | CCCTTGCGGAAGTTGCTTCA |
| Amplicon primer *Lrrc8d* RV | CAGCTCCTGCTTATCCTGGG |
| Primer for TIDE amplicon *Lrrc8a* sgRNA3 FW | GGCCATTGGTGGGGTTCTTA |
| Primer for TIDE amplicon *Lrrc8a* sgRNA3 RV | GGTGCGTGGAAACTTGAACC |
| Primer for TIDE *Lrrc8a* sgRNA3 sequencing FW | ACAGAGCTCCGCTACTTTGC |
| Primer for TIDE amplicon *Lrrc8d* sgRNA3 FW | AAAGGGTTCTCATTGGTCCCAC |
| Primer for TIDE amplicon *Lrrc8d* sgRNA3 RV | CGCCTTAGTTGTCCAGGGAG |
| Primer for TIDE *Lrrc8d* sgRNA3 sequencing FW | AGGGAGGGCCAGATGGTAAC |
| Inverse PCR pOZ-N-FH-IL2Rα FW | TCGAGAGATCCGGGAGACACAA |
| Inverse PCR pOZ-N-FH-IL2Rα RV | CTCGAGCGGAAGATCTGGCAGTCT |
| cDNA amplification *Lrrc8a* FW | GATCTTCCGCTCGAGATGATTCCGGTGACAGAGCTCCGC |
| cDNA amplification *Lrrc8a* RV | TTGTGTCTCCCGGATCTCTCGATGCGGCCCTACAGATCCTCTTCTGAGAT­GAGTTTTTGTTCTCCTCCAGCGGCCGCGGCCTGCTCCTTGTCAGCTC |
| cDNA amplification *Lrrc8d* FW | GATCTTCCGCTCGAGATGTTTACCCTTGCGGAAGTTGC |
| cDNA amplification *Lrrc8d* RV | TTGTGTCTCCCGGATCTCTCGATGCGGCCCTACAGATCCTCTTCTGAGAT­GAGTTTTTGTTCTCCTCCAGCGGCCGCAATCCCGTTTGCAAAGGGGACA |
| *Lrrc8d* knockout mice genotyping primer deletion PCR FW | TTTCAGGAATGTTTACCCTTGCGG |
| *Lrrc8d* knockout mice genotyping primer deletion PCR RV | TGCATCGTGTCCTGTTTAAAGGGC |
| *Lrrc8d* knockout mice genotyping primer wild type PCR FW | TTTCAGGAATGTTTACCCTTGCGG |
| *Lrrc8d* knockout mice genotyping primer wild type PCR RV | GGTGTGTGGCTGTTTCCATCCTG |
| *Lrrc8d* knockout mice RTqPCR primer pair 1 FW | CTGCCTCTACACTCTCTTCTGGC |
| *Lrrc8d* knockout mice RTqPCR primer pair 1 RV | CGCAAAGTCGTTCTTGACATCCG |
| *Lrrc8d* knockout mice RTqPCR primer pair 2 FW | CCTAAGATGAGCGCAAGTTGAA |
| *Lrrc8d* knockout mice RTqPCR primer pair 2 RV | CCACAGGACTAGAACACCTGCTAA |
| *Hprt* primer FW | CCTAAGATGAGCGCAAGTTGAA |
| *Hprt* primer RV | CCACAGGACTAGAACACCTGCTAA |
