# Supplementary material for: Loss of the Volume-regulated Anion Channel Components LRRC8A and LRRC8D Limits Platinum Drug Efficacy
Source: Cancer Res Commun. 2022 Oct 26;2(10):1266–81. doi: 10.1158/2767-9764.CRC-22-0208 (PMC7613873; doi:10.1158/2767-9764.CRC-22-0208)
Supplement: Figure FS1 — Generation of monoclonal knockout cell lines of Lrrc8a or Lrrc8d used in main Figure [file crc-22-0208-s03.docx]

**Figure S1**

**
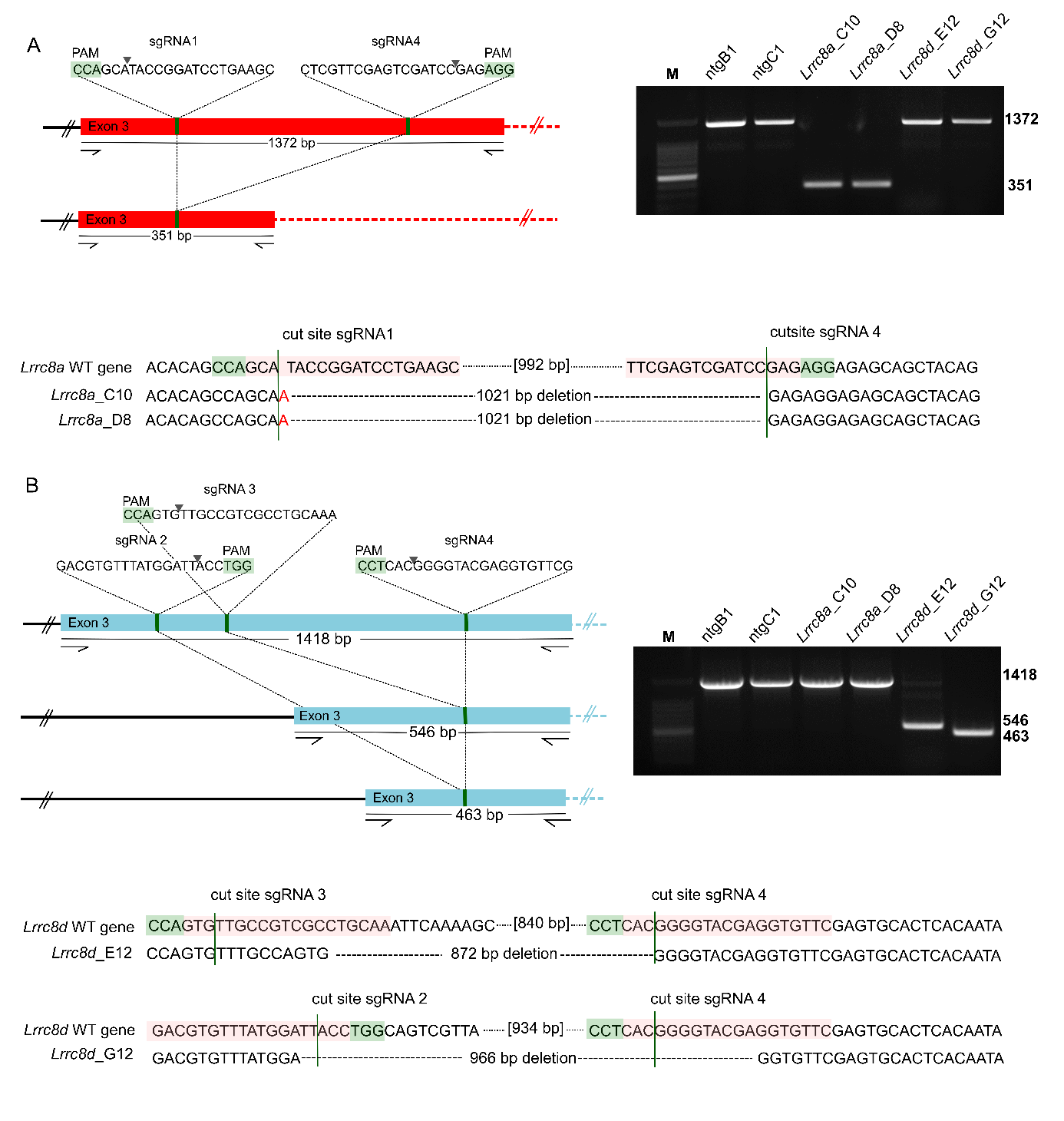
**

**Figure S1. Generation of monoclonal knockout cell lines of *Lrrc8a* or *Lrrc8d* used in main Figure 1 A)** Layout of the paired gRNA position targeting *Lrrc8a* and design of the PCR spanning the targeted gene region. The PCR amplification identified clonal *Lrrc8a* KO cell lines (C10 and D8) with large exon 3 deletions that also create an additional frame shift as confirmed by DNA sequencing. **B)** Layout of the paired gRNA position targeting *Lrrc8d* and design of the PCR to amplify and sequence the exon 3 region. Two clonal cell lines (E12 and G12) with large deletions in *Lrrc8d* were identified.
