# Supplementary material for: Loss of the Volume-regulated Anion Channel Components LRRC8A and LRRC8D Limits Platinum Drug Efficacy
Source: Cancer Res Commun. 2022 Oct 26;2(10):1266–81. doi: 10.1158/2767-9764.CRC-22-0208 (PMC7613873; doi:10.1158/2767-9764.CRC-22-0208)
Supplement: Figure FS2 — Blasticidin and Pt treatment of Lrrc8a or Lrrc8d rescue cell lines data associated with main Figure 1 [file crc-22-0208-s04.docx]

**Figure S2**

**
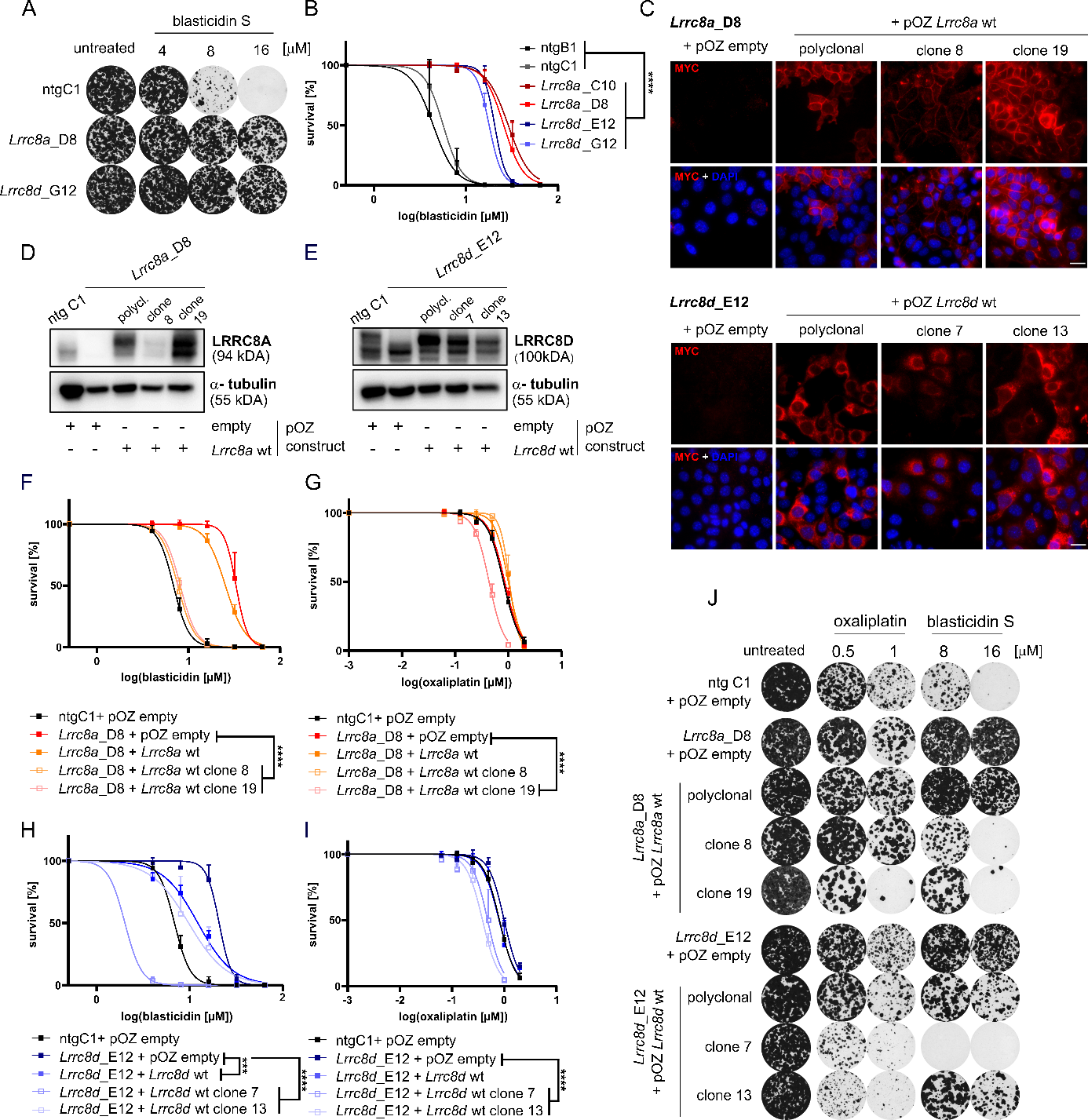
**

**Figure S2. Blasticidin and Pt treatment of *Lrrc8a* or *Lrrc8d* rescue cell lines data associated with main Figure 1 A)** Representative images of clonogenic survival assay using wild type and LRRC8A/D-deficient cell lines treated with blasticidin S. **B)** Quantification of clonogenic growth assays using wild type and LRRC8A/D-deficient cell lines in the presence of blasticidin S. Data represent mean ± SD of three independent replicates and were fitted to a four parameter logistic (4PL) sigmoidal curve. *P*-values are calculated by one-way ANOVA followed by Tukey's multiple comparisons test for the log(IC50) values of the survival curves *****p*<0.0001. **C)** Representative immunofluorescence images of the C-terminally Myc tagged *Lrrc8a* or *Lrrc8d* rescue constructs expressed in either *Lrrc8a*_D8 or *Lrrc8d*_E12 monoclonal knockout cell lines. The scale bar represents 20µm **D+E)** Representative Western blots of *Lrrc8a* or *Lrrc8d* rescue construct expression in the polyclonal or monoclonal lines used for clonogenic growth assays. **F-I)** Quantification of clonogenic growth assays using the different *Lrrc8a* (*Lrrc8a*_D8 + pOZ empty, + pOZ *Lrrc8a* wt polyclonal or clonal lines) or *Lrrc8d* (*Lrrc8d*_D8 + pOZ empty, + pOZ *Lrrc8d* wt polyclonal or clonal lines) rescue cell lines treated with blasticidin S or oxaliplatin. As negative controls, empty vector-transduced cell lines were used. Data represent mean ± SD of three independent replicates and were fitted to a four parameter logistic (4PL) sigmoidal curve. *P*-values are calculated by one-way ANOVA followed by Tukey's multiple comparisons test for the log(IC50) values of the survival curves. *****p*<0.0001, ****p*<0.001. **J)** Representative images of selected conditions of the clonogenic growth assays in the presence of oxaliplatin or blasticidin S, using the rescue cell lines from **F-I** are shown.
