# Supplementary material for: Loss of the Volume-regulated Anion Channel Components LRRC8A and LRRC8D Limits Platinum Drug Efficacy
Source: Cancer Res Commun. 2022 Oct 26;2(10):1266–81. doi: 10.1158/2767-9764.CRC-22-0208 (PMC7613873; doi:10.1158/2767-9764.CRC-22-0208)
Supplement: Figure FS3 — Pt-drug treatment of polyclonal cell lines with wild type as well as Lrrc8a or Lrrc8d knockout alleles shows positive selection for knockout alleles [file crc-22-0208-s05.docx]

**Figure S3**

**
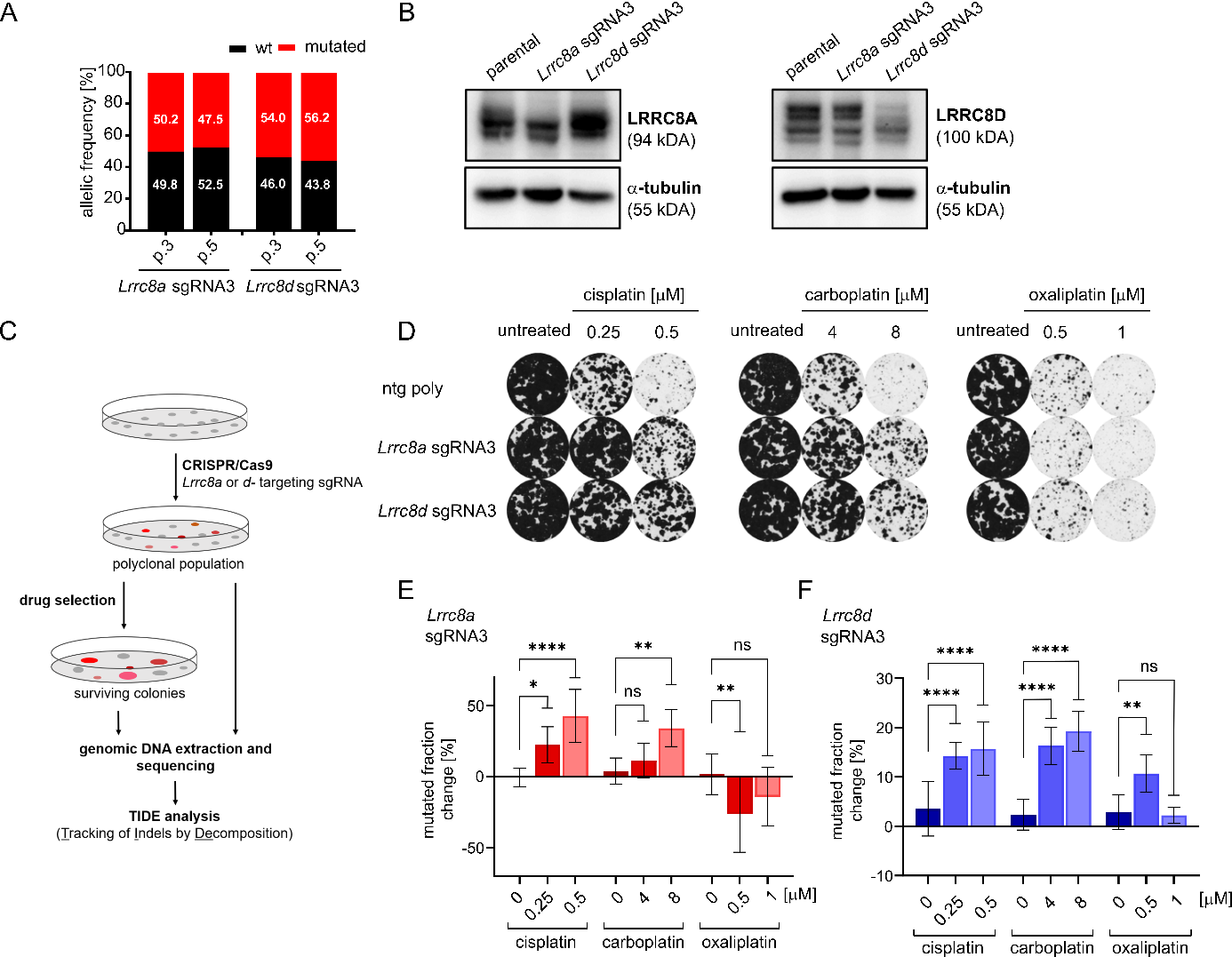
**

**Figure S3 Pt-drug treatment of polyclonal cell lines with wild type as well as *Lrrc8a* or *Lrrc8d* knockout alleles shows positive selection for knockout alleles A)** Allelic frequency distribution of wt and mutant alleles in polyclonal cell pools after transfection with either *Lrrc8a* (sgRNA3) or *Lrrc8d* (sgRNA3) targeting sgRNAs over the course of two passages. **B)** LRRC8A or LRRC8D protein expression levels in the parental and polyclonal knock out cell lines. **C)** Experimental layout of the competition assay using the polyclonal cell pools. After drug selection, the genomic DNA is harvested, sequenced and analyzed by Tracking of Indels by Decomposition (TIDE) analysis. **D)** Selected wells from the competition assays using the polyclonal cell pools, which were treated with either cisplatin, carboplatin, or oxaliplatin**. E-F)** Change in percent of the allelic frequency of mutations in *Lrrc8a* or *Lrrc8d* within the polyclonal cell pools after treatment with two concentrations of cisplatin, carboplatin or oxaliplatin*.* Percentages of wild type and mutated alleles of each population after treatment were determined by TIDE analysis. The results of six replicates are shown. Significance is determined by two-way ANOVA (followed by Dunnett's multiple comparisons test). *****p*<0.0001, ***p*<0.01, **p*<0.05.
