# Supplementary material for: Loss of the Volume-regulated Anion Channel Components LRRC8A and LRRC8D Limits Platinum Drug Efficacy
Source: Cancer Res Commun. 2022 Oct 26;2(10):1266–81. doi: 10.1158/2767-9764.CRC-22-0208 (PMC7613873; doi:10.1158/2767-9764.CRC-22-0208)
Supplement: Figure FS4 — TIDE analysis and tumor growth curves of Kaplan Meyer survival graphs shown in main Figure 3 [file crc-22-0208-s06.docx]

**Figure S4**

**
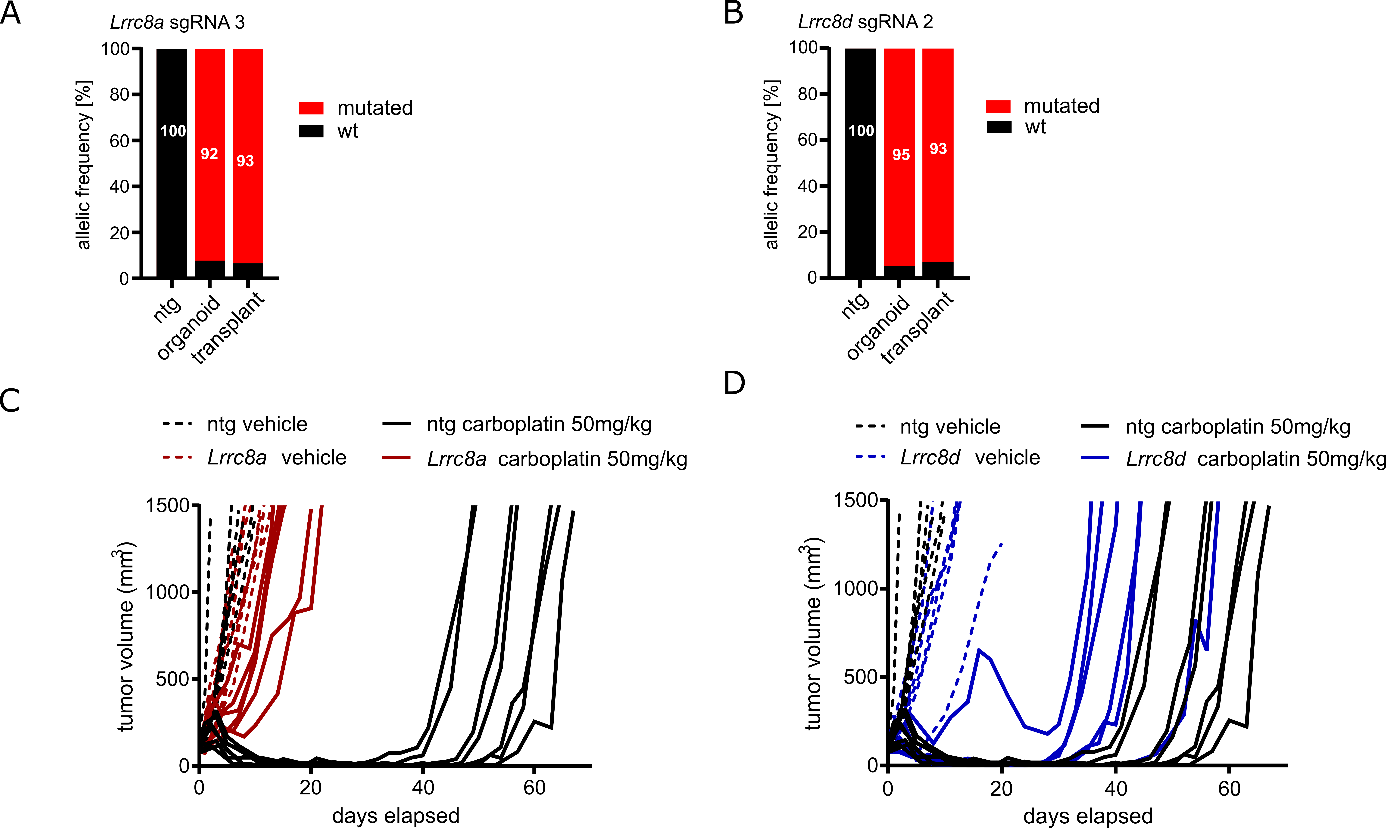
**

**Figure S4 TIDE analysis and tumor growth curves of Kaplan Meyer survival graphs shown in main Figure 3 A-B)** Allelic frequency distribution in original organoid culture transduced by either gRNA3 targeting *Lrrc8a* or gRNA2 targeting *Lrrc8d* and the tumor, which was selected for transplantation. **C-D)** Tumor growth curves of wild type, LRRC8A, or LRRC8D-deficient tumors treated with two cycles of 50 mg/kg carboplatin. In both graphs, the same wild type (ntg) tumors are shown.
