# Supplementary material for: Loss of the Volume-regulated Anion Channel Components LRRC8A and LRRC8D Limits Platinum Drug Efficacy
Source: Cancer Res Commun. 2022 Oct 26;2(10):1266–81. doi: 10.1158/2767-9764.CRC-22-0208 (PMC7613873; doi:10.1158/2767-9764.CRC-22-0208)
Supplement: Figure FS5 — Lrrc8d KO mice tolerate higher cisplatin doses than wild type mice [file crc-22-0208-s07.docx]

**Figure S5**

**
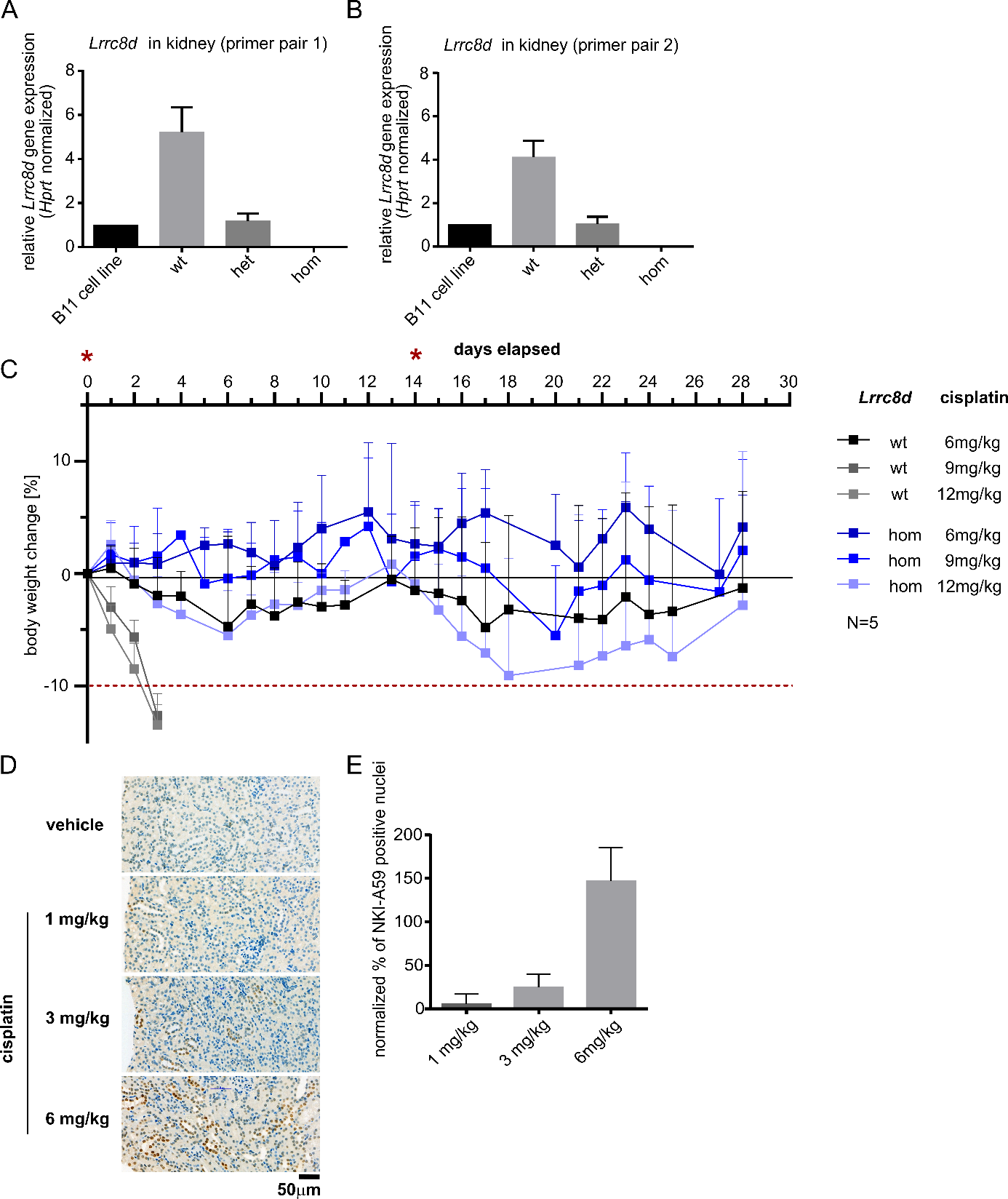
**

**Figure S5 *Lrrc8d* KO mice tolerate higher cisplatin doses than wild type mice** **A-B)** *Lrrc8d* expression levels in a B11 control cell line, wild type, heterozygous and homozygous KO mice kidneys determined by two different primer pairs. **C)** Body weight percent change of wild type mice treated with two cycles of 6 mg/kg of cisplatin and homozygous *Lrrc8d* KO mice treated with two cycles of 6, 9, or 12 mg/kg of cisplatin. The asterisks indicate the treatment time points. Each group consisted of 5 animals. **D)** Representative images of the cisplatin adduct staining in the kidney cortex of wild type FVB/N mice treated with increasing concentrations of cisplatin using the anti-cisplatin adduct antibody NKI-A59. The kidneys were harvested after 6 hours of treatment. The scale bar represents 50µm. **E)** Quantification of positive nuclei per total number of cells of 22 images at 40x magnification of the cortical kidney region of each mouse (untreated N=3, 1 mg/kg N=1, 3 mg/kg N=1, 6 mg/kg N=2).
