# Supplementary material for: Loss of the Volume-regulated Anion Channel Components LRRC8A and LRRC8D Limits Platinum Drug Efficacy
Source: Cancer Res Commun. 2022 Oct 26;2(10):1266–81. doi: 10.1158/2767-9764.CRC-22-0208 (PMC7613873; doi:10.1158/2767-9764.CRC-22-0208)
Supplement: Figure FS6 — Analysis of publicly available ovarian cancer dataset to identify the association of LRRC8A or LRRC8D expression with outcome of chemotherapy with cisplatin [file crc-22-0208-s08.docx]

**Figure S6**

**
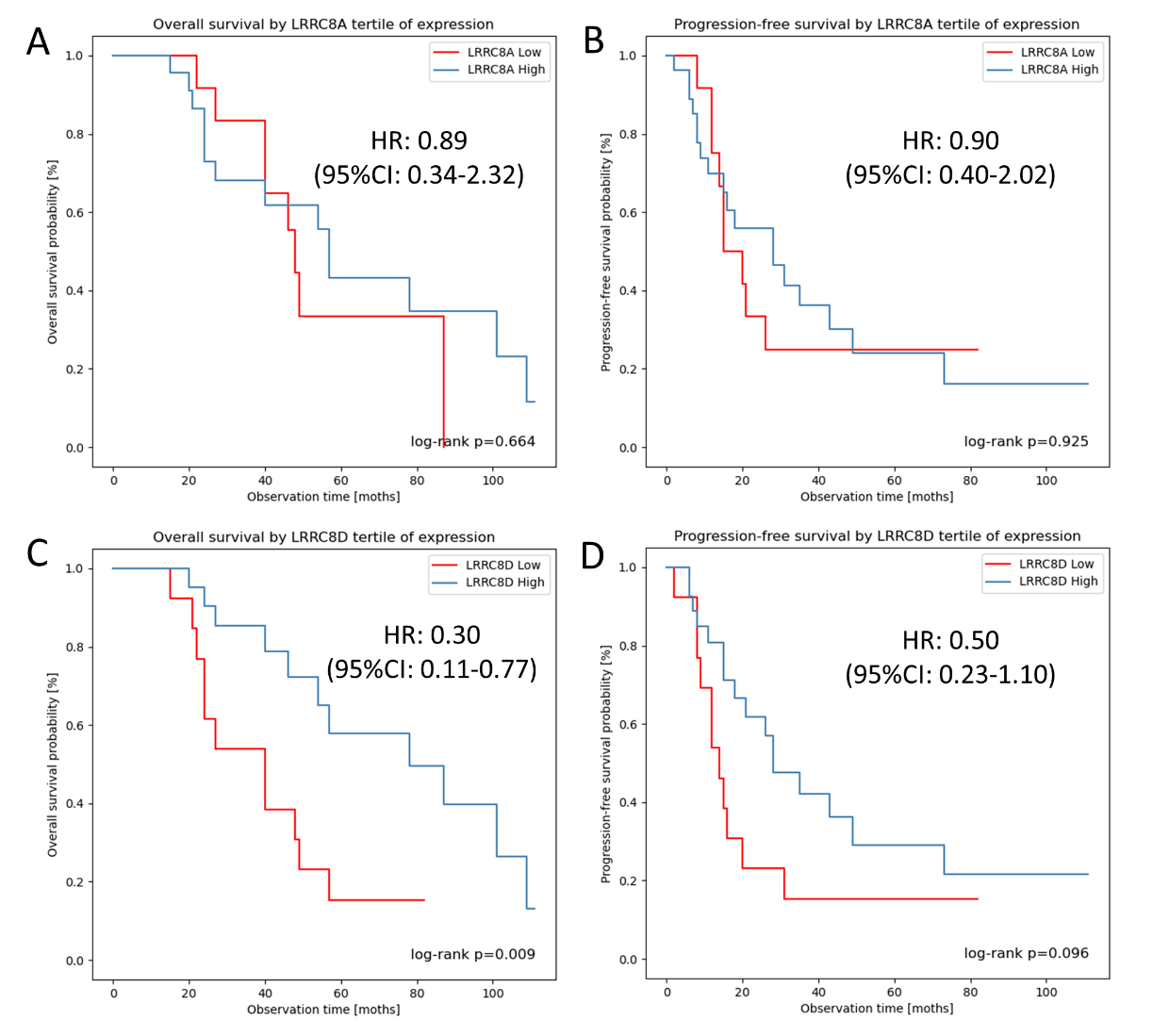
**

**Figure S6 Analysis of publicly available ovarian cancer dataset to identify the association of *LRRC8A* or *LRRC8D* expression with outcome of chemotherapy with cisplatin. A+C)** Overall survival of patients from GSE32063 study with advanced stage high-grade serous ovarian cancer treated with Platinum-taxanes standard chemotherapy categorized by tertile of expression of *LRRC8A* or *LRRC8D*. **B+D)** Progression-free survival of patients from the GSE32063 study categorized by a tertile of expression of *LRRC8A* or *LRRC8D*.
